# Prion protein signaling induces M2 macrophage polarization and protects from lethal influenza infection in mice

**DOI:** 10.1371/journal.ppat.1008823

**Published:** 2020-08-26

**Authors:** Junji Chida, Hideyuki Hara, Keiji Uchiyama, Etsuhisa Takahashi, Hironori Miyata, Hidetaka Kosako, Yukiko Tomioka, Toshihiro Ito, Hiroyuki Horiuchi, Haruo Matsuda, Hiroshi Kido, Suehiro Sakaguchi

**Affiliations:** 1 Division of Molecular Neurobiology, The Institute for Enzyme Research (KOSOKEN), Tokushima University, Tokushima, Japan; 2 Division of Enzyme Chemistry, The Institute for Enzyme Research, Tokushima University (KOSOKEN), Tokushima, Japan; 3 Animal Research Center, School of Medicine, University of Occupational and Environmental Health, Kitakyushu, Japan; 4 Division of Cell Signaling, Fujii Memorial Institute of Medical Sciences, Tokushima University, Kuramoto-cho, Tokushima, Japan; 5 Laboratory of Laboratory Animal Science, Joint Department of Veterinary Medicine, Faculty of Agriculture, Tottori University, Tottori, Japan; 6 Avian Zoonosis Research Center, Faculty of Agriculture, Tottori University, Koyama-cho, Tottori, Japan; 7 Laboratory of Immunobiology, Graduate School of Integrated Sciences for Life, Hiroshima University, Japan; 8 Laboratory of Immunobiology, Department of Molecular and Applied Bioscience, Graduate School of Biosphere Science, Hiroshima University, Japan; University of Minnesota, UNITED STATES

## Abstract

The cellular prion protein, PrP^C^, is a glycosylphosphatidylinositol anchored-membrane glycoprotein expressed most abundantly in neuronal and to a lesser extent in non-neuronal cells. Its conformational conversion into the amyloidogenic isoform in neurons is a key pathogenic event in prion diseases, including Creutzfeldt-Jakob disease in humans and scrapie and bovine spongiform encephalopathy in animals. However, the normal functions of PrP^C^ remain largely unknown, particularly in non-neuronal cells. Here we show that stimulation of PrP^C^ with anti-PrP monoclonal antibodies (mAbs) protected mice from lethal infection with influenza A viruses (IAVs), with abundant accumulation of anti-inflammatory M2 macrophages with activated Src family kinases (SFKs) in infected lungs. A SFK inhibitor dasatinib inhibited M2 macrophage accumulation in IAV-infected lungs after treatment with anti-PrP mAbs and abolished the anti-PrP mAb-induced protective activity against lethal influenza infection in mice. We also show that stimulation of PrP^C^ with anti-PrP mAbs induced M2 polarization in peritoneal macrophages through SFK activation *in vitro* and *in vivo*. These results indicate that PrP^C^ could activate SFK in macrophages and induce macrophage polarization to an anti-inflammatory M2 phenotype after stimulation with anti-PrP mAbs, thereby eliciting protective activity against lethal infection with IAVs in mice after treatment with anti-PrP mAbs. These results also highlight PrP^C^ as a novel therapeutic target for IAV infection.

## Author summary

We show here that stimulation of PrP^C^ with anti-PrP mAbs protected mice from lethal infection with IAVs by stimulating macrophage polarization to an anti-inflammatory M2 phenotype through activation of SFKs in infected lungs. IAVs are causative agents for seasonal epidemic outbreaks of influenza, and often cause high morbidity and mortality in infected people, particularly in the young and elderly and those with underlying chronic diseases. It has also been reported that highly pathogenic avian IAVs have the potential to infect humans and cause high morbidity and mortality in infected individuals. The other problem with IAV infections is that new IAV strains, which are resistant to currently available anti-influenza agents such as neuraminidase inhibitors, have been emerging in human populations. Our current results showing that targeting PrP^C^ with anti-PrP mAbs protected against lethal infection with IAVs in mice give rise to the possibility that PrP^C^-targeting therapeutics might be beneficial in influenza infection. Moreover, since PrP^C^ is a host molecule, PrP^C^-targeting drugs might not induce drug-resistant IAVs. Thus, PrP^C^ might be a novel target molecule for therapeutic development for IAV infection, although further studies are needed.

## Introduction

The normal cellular isoform of prion protein, designated PrP^C^, is a membrane glycoprotein tethered to the outer cell membrane via a glycosylphosphatidylinositol (GPI) anchor moiety and expressed most abundantly in the brain, particularly by neurons, and to a lesser extent in non-neuronal tissues such as heart, lung, and spleen [1–3]. Conformational conversion of PrP^C^ into the amyloidogenic isoform, PrP^Sc^, in the brain is a key pathogenic event in prion diseases, a group of neurodegenerative disorders, which include Creutzfeldt-Jakob disease in humans and scrapie and bovine spongiform encephalopathy in animals [1]. Mice devoid of PrP^C^ (*Prnp*^*0/0*^) have been shown to be vulnerable to ischemic injury in the brain, heart and kidney, with enhanced apoptosis in the injured tissues [4–8], suggesting that PrP^C^ could have a cellular protective function in these tissues. It has also been previously shown that lung epithelial cells in *Prnp*^*0/0*^ mice were highly vulnerable to apoptotic cell death after infection with influenza A viruses (IAVs), suggesting that PrP^C^ could have a protective role for lung epithelial cells [3,9].

IAVs are enveloped, negative sense, single-stranded RNA viruses causing seasonal epidemic outbreaks of the acute upper respiratory disease influenza [10]. Severe influenza infections are often lethal, causing high morbidity and mortality in infected people, particularly in the young and elderly and those with underlying chronic diseases in lung or cardiovascular systems [10]. Currently available main anti-influenza agents are viral protein-targeting agents such as neuraminidase inhibitors. However, emergence of influenza viruses that are resistant to these agents has been reported and may become a serious problem if these drug-resistant IAVs spread in human populations [11–13]. It has also been shown that highly pathogenic avian IAVs of the H5N1 and H7N9 subtypes have the potential to infect humans, causing high morbidity and mortality in infected individuals, raising another concern about the pandemic potential of these avian viruses in human populations [14–16]. Therefore, identification of new target molecules for anti-influenza agents is urgently awaited.

In this study, we show a novel function of PrP^C^ to induce anti-inflammatory M2 macrophage polarization through activation of Src family kinases (SFKs), and that anti-PrP monoclonal antibodies (mAbs) stimulated the novel activity of PrP^C^ inducing M2 macrophages and thereby protected mice from lethal infection with IAVs. These results suggest that PrP^C^ could be a novel therapeutic target for influenza infection by promoting M2 macrophage polarization.

## Results

### 38–2 anti-PrP mAb provides protection against lethal IAV infection in mice

**Fig 1:**
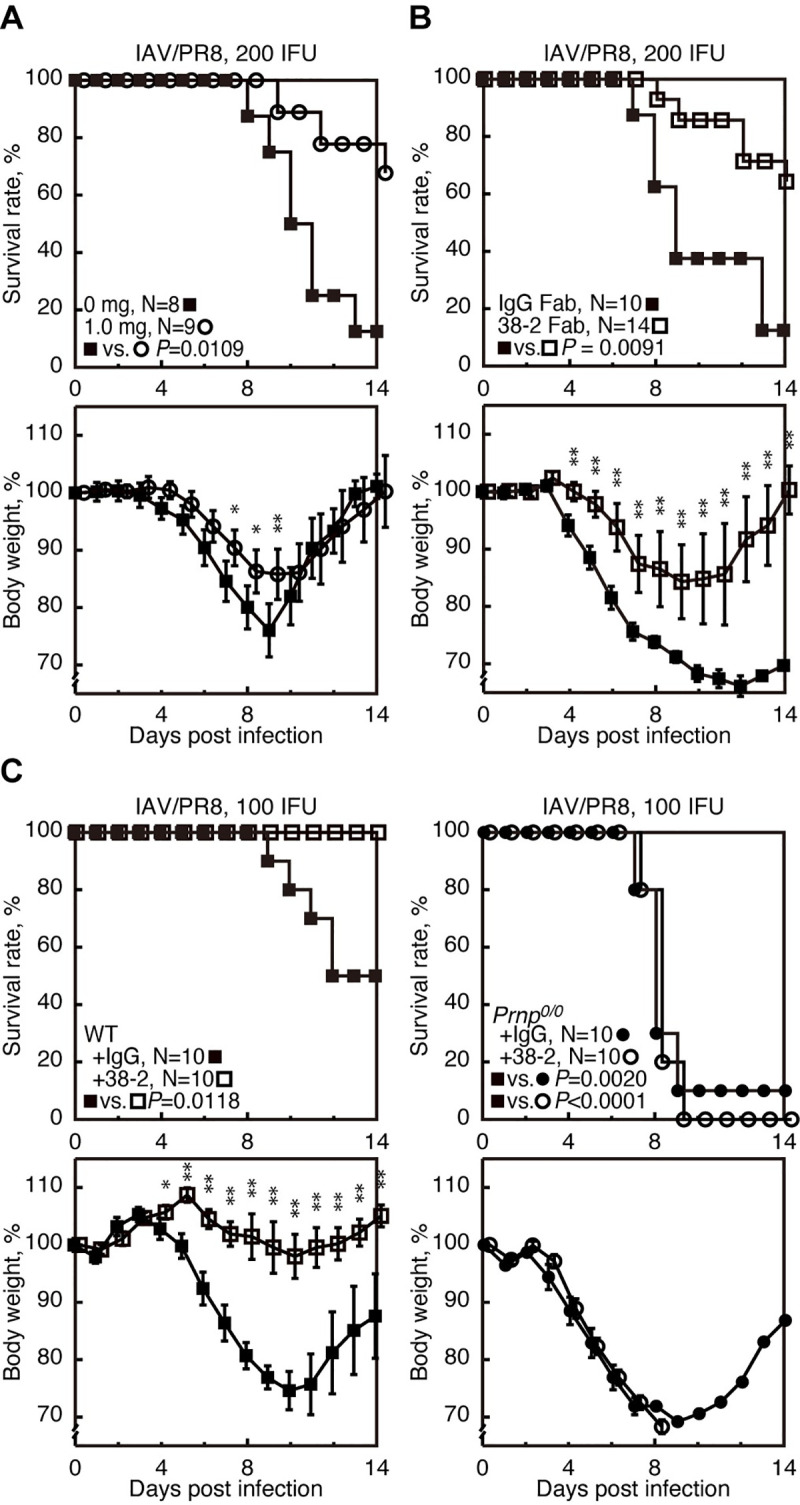
38–2 mAb provokes protection against lethal infection with IAV/PR8 in mice. (**A**) The survival rate (%, upper panel) and body weight loss (%, lower panel) of WT mice intraperitoneally administered with either the buffer alone (0 mg/mouse) or 38–2 mAb (1mg/mouse) 1 day before intranasal infection with 200 IFU of IAV/PR8. Error bars, standard deviations (SD). (**B**) Survival rate (%, upper panel) and body weight loss (%, lower panel) of WT mice intraperitoneally administered with control IgG Fab and 38–2 Fab fragments 1 day before intranasal infection with 200 IFU of IAV/PR8. Error bars, SD. (**C**) Survival rate (%, upper panel) and body weight loss (%, lower panel) of WT and *Prnp*^*0/0*^ mice intraperitoneally administered with 38–2 mAb and control IgG 1 day before intranasal infection with 100 IFU of IAV/PR8. Error bars, SD.*, p<0.05; **, p<0.01.

To examine if PrP^C^ could be stimulated by anti-PrP Abs and provide beneficial effects in IAV infection, various doses of 38–2 anti-PrP IgG mAb, which recognizes residues 41–45 of mouse PrP^C^ [17], was intraperitoneally administered to C57BL/6 wild-type (WT) mice 1 day before intranasal infection with influenza virus strain A/Puerto Rico/8/34 (H1N1) (hereafter referred to as IAV/PR8) with 200 infectious units (IFU). The 38–2 hybridoma was established by fusion of myeloma cells with splenocytes from *Prnp*^*0/0*^ mice immunized with recombinant mouse PrP [17]. Approximately 90% of control mice, which received vehicle buffer alone, died by 14 days post infection (dpi) **(Fig 1A and S1 Fig)**. However, 38–2 mAb markedly reduced the mortality of infected mice in a dose-dependent manner **(Fig 1A and S1 Fig)**. Weight loss was also mitigated in 38–2 mAb-treated, IAV/PR8-infected mice compared to control mice **(Fig 1A and S1 Fig)**. These results indicate that targeting of PrP^C^ with 38–2 mAb could confer protection against lethal IAV infection in mice. We then investigated the Fab fragment of 38–2 mAb for its protective activity against lethal IAV infection, by intraperitoneally injecting the 38–2 Fab fragment (1 mg/mouse) into WT mice 1 day before infection with 200 IFU of IAV/PR8. The 38–2 Fab fragment also significantly reduced the mortality of infected WT mice compared to control Fab fragment **(Fig 1B)**, indicating that the 38–2 mAb-mediated protective activity of PrP^C^ against lethal IAV infection does not depend on cross-linking of PrP^C^ or any Fc-dependent Ab functions.

To confirm that the protective activity of 38–2 mAb against lethal IAV infection is mediated through targeting PrP^C^, we similarly injected 38–2 mAb (1 mg/mouse) into *Prnp*^*0/0*^ and WT mice 1 day before infection with 100 IFU of IAV/PR8. Purified unimmunized mouse polyclonal IgG was also administered into WT mice as a control. Since *Prnp*^*0/0*^ mice had been reported to be highly susceptible to IAV/PR8 infection [3], we used lower doses (100 IFU) of IAV/PR8 in this experiment. 38–2 mAb consistently reduced the mortality and weight loss of infected WT mice **(Fig 1C)**. However, the mortality and weight loss of infected *Prnp*^*0/0*^ mice was not affected by 38–2 mAb **(Fig 1C)**. These results confirm that targeting of PrP^C^ is essential for 38–2 mAb to elicit its protective activity against lethal IAV infection.

### 38–2 mAb suppresses IAV-infected lung pathologies

**Fig 2:**
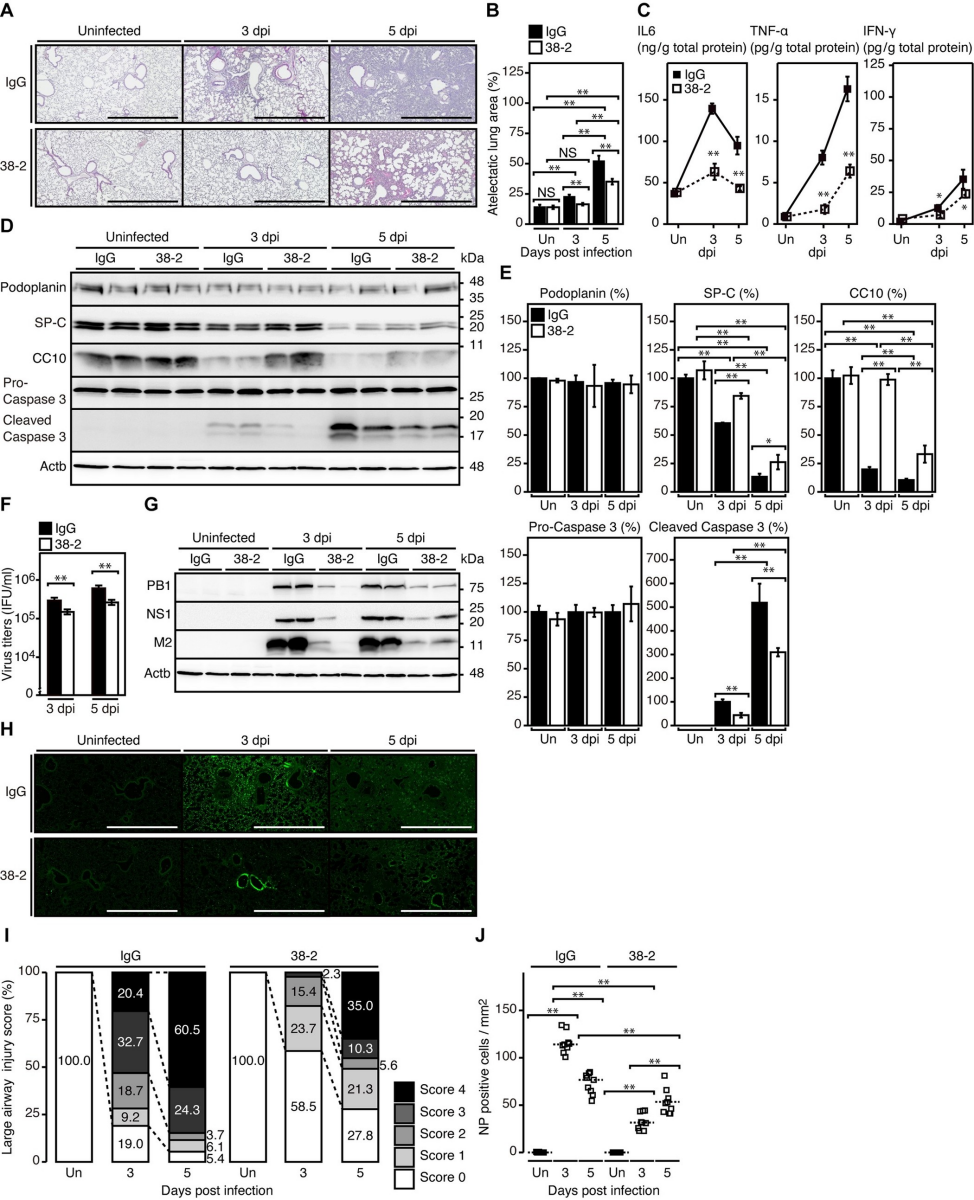
38–2 mAb suppresses lung pathologies of IAV-infected mice. **(A**) Hemotoxylin-Eosin-stained lungs from control IgG- and 38–2 mAb-treated mice uninfected and at 3 and 5 dpi with 200 IFU of IAV/PR8. Bar, 1 mm. Atelectatic area (%) (**B**) and levels of pro-inflammatory cytokines (**C**) in the lungs from control IgG- and 38–2 mAb-treated mice uninfected and at 3 and 5 dpi with 200 IFU of IAV/PR8 (n = 3 in each group). Un, uninfected. (**D**) Western blotting for the AT1 cell marker podoplanin, the AT2 cell marker SP-C, the Clara cell marker CC10, pro-caspase 3, and the cleaved caspase 3 in lungs from control IgG- and 38–2 mAb-treated mice uninfected and at 3 and 5 dpi with 200 IFU of IAV/PR8. Actb, β-actin. (**E**) The percent density of indicated proteins against the densities of the corresponding proteins in IgG-treated, uninfected lungs after being normalized by the densities of β-actin (n = 3 in each group). Un, uninfected. (**F**) Virus titers in the lungs from control IgG- and 38–2 mAb-treated mice at 3 and 5 dpi with 200 IFU of IAV/PR8 (n = 3 in each group). (**G**) Western blotting for the viral proteins, PB1, NS1, and M2 in the lungs from control IgG- and 38–2 mAb-treated mice uninfected and at 3 and 5 dpi with 200 IFU of IAV/PR8. Actb, β-actin. (**H**) Immunofluorescent staining for the viral protein NP in the lungs from control IgG- and 38–2 mAb-treated mice uninfected and at 3 and 5 dpi with 200 IFU of IAV/PR8. Bar, 1 mm. (**I**) Injury score (%) of NP-positive large airways in (**H**). Score 0, intact epithelial cells in the airway without NP-positive cells; score 1, intact epithelial cells in the airway with <50% NP-positive cells; score 2, intact epithelial cells in the airway with >50% NP-positive cells; score 4, <50% epithelial cell injuries in the airway with NP-positive cells; score 5, >50% epithelial cell injuries in the airway with NP-positive cells. n = 3 slices × 3 mice in each group. (**J**) NP-positive cells/mm^2^ in the lung parenchymal areas in (**H**). n = 3 slices × 3 mice in each group.*, p<0.05; **, p<0.01.

To investigate the effects of 38–2 mAb on IAV-infected lung pathologies, we microscopically examined the lungs of control IgG- and 38–2 mAb-treated WT mice uninfected and at 3 and 5 dpi with IAV/PR8 (200 IFU). No solid lesions with infiltration of inflammatory cells were detected in the parenchymal and bronchiolar areas of uninfected lungs **(Fig 2A)**. However, inflammatory cells had infiltrated these areas of control IgG- and 38–2 mAb-treated, IAV/PR8-infected lungs, particularly markedly at 5 dpi, with much milder infiltration of inflammatory cells in 38–2 mAb-treated, IAV/PR8-infected lungs than in control lungs at 3 and 5 dpi **(Fig 2A)**. Neither hyaline membrane formation nor hemorrhage was observed in these lungs **(Fig 2A)**. Wet weight of 38–2 mAb-treated, IAV/PR8-infected lungs was significantly lighter than that of control lungs **(S2 Fig)**, suggesting lower exudates in the former lungs than in the latter. Atelectatic lung areas were also smaller in the former lungs than in the latter **(Fig 2B)**. Levels of inflammatory cytokines such as interleukin-6 (IL-6), tumor necrosis factor-α (TNF-α), and interferon-γ (IFN-γ) were also lower in 38–2 mAb-treated, IAV/PR8-infected lungs than in control lungs **(Fig 2C)**. We also assessed lung damage in 38–2 mAb-treated, IAV/PR8-infected mice. Apoptotic marker cleaved caspase 3 fragments were lower in 38–2 mAb-treated, IAV/PR8-infected lungs than in control lungs **(Fig 2D, 2E and S3 Fig)**. Terminal deoxynucleotidyl transferase-mediated dUTP nick-end labeling (TUNEL) assay also showed lower TUNEL-positive cells in 38–2 mAb-treated, IAV/PR8-infected lungs than in control lungs **(S4 Fig)**. Protein levels of the alveolar type 1 (AT1) epithelial cell marker podoplanin was unchanged during IAV infection **(Fig 2D, 2E and S3 Fig)**. This is consistent with AT1 epithelial cells in C57BL/6 mice being resistant to IAV infection [18]. In contrast, pulmonary surfactant protein C (SP-C) and Clara cell 10-kDa protein (CC10), which are specific markers for AT2 and Clara epithelial cells, respectively, remained higher in 38–2 mAb-treated, IAV/PR8-infected lungs than in control lungs **(Fig 2D, 2E and S3 Fig)**. This suggests that AT2 and Clara cells are less damaged in 38–2 mAb-treated, IAV/PR8-infected lungs than in control lungs. Virus titers and levels of viral proteins including PB1, NS1 and M2 were also lower in 38–2 mAb-treated, IAV/PR8-infected lungs than in control lungs **(Fig 2F and 2G)**. Viral protein NP-positive areas were also limited in the former lungs compared to the latter **(Fig 2H)**. The NP-positive cells were observed in the airway and parenchymal areas of both IgG- or 38–2 mAb-treated, IAV/PR8-infected lungs at 3 and 5 dpi. However, they were gradually increased in the airway and parenchymal areas of 38–2 mAb-treated lungs, but rapidly in control lungs **(Fig 2I and 2J)**. These results suggest that 38–2 mAb also could affect the susceptibility of lung epithelial cells to IAV/PR8 without altering the cell tropism of IAV/PR8 in the lung. Taken together, these results indicate that 38–2 mAb could suppress IAV infection-induced lung pathologies, including inflammatory cell infiltration, inflammatory cytokine production, epithelial cell damage, and IAV production.

### 38–2 mAb does not activate SOD1 in IAV-infected lungs

**Fig 3:**
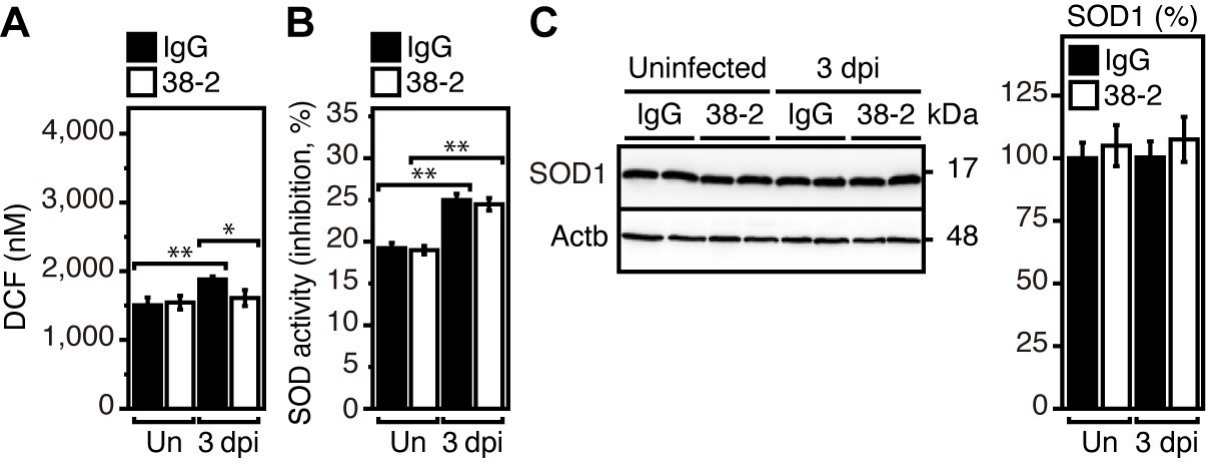
SOD1 is not activated by 38–2 mAb in IAV-infected lungs. DCF levels representing ROS levels (**A**) and SOD activity (**B**) in the lungs of IgG- and 38–2 mAb-treated mice uninfected and at 3 dpi with 200 IFU of IAV/PR8 (n = 3 in each group). Un, uninfected. (**C**) Western blotting for SOD1 in the lungs from control IgG- and 38–2 mAb-treated mice uninfected and at 3 dpi with 200 IFU of IAV/PR8. Actb, β-actin. The percent density of SOD1 against the densities of SOD1 in IgG-treated, uninfected lungs after being normalized by the densities of β-actin (n = 3 in each group). Un, uninfected. *, p<0.05; **, p<0.01.

It has been reported that PrP^C^ could provide protection against IAV infection in mice, by reducing reactive oxygen species (ROS) through activation of Cu/Zn-dependent superoxide dismutase (SOD1) in infected lungs [3]. To address if SOD1 activity could be involved in the 38–2 mAb-induced protective activity of PrP^C^ against lethal IAV infection, we investigated whether 38–2 mAb could induce activation of SOD1 in IAV-infected lungs. ROS levels were slightly lower in 38–2 mAb-treated lungs than in control lungs at 3 dpi with IAV/PR8 (200 IFU) **(Fig 3A)**. However, enzymatic activity of SOD1 was similarly elevated in both lungs **(Fig 3B)**. Protein levels of SOD1 were also unchanged in both lungs **(Fig 3C)**. These results suggest that the SOD1-mediated anti-oxidative activity of PrP^C^ is not involved in the 38–2 mAb-induced protective activity of PrP^C^ against lethal IAV infection.

### SFK activation is essential for 38–2 mAb to elicit the protective activity against lethal IAV infection

**Fig 4:**
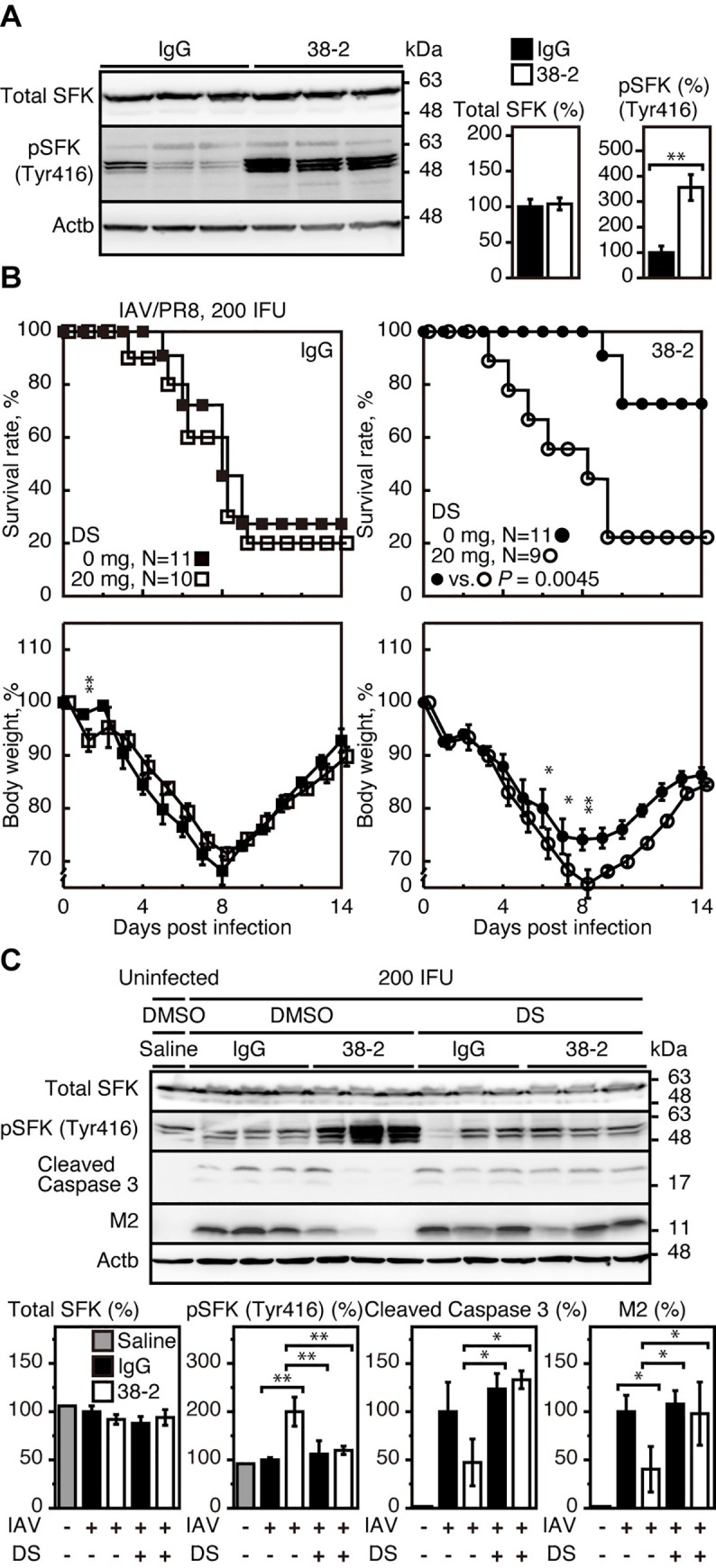
38–2 mAb activates SFK in IAV-infected lungs and elicits protective activity against lethal IAV infection. (**A**) Western blotting (left panel) for total and phosphorylated SFK in the lungs from control IgG- and 38–2 mAb-treated mice at 3 dpi with 200 IFU of IAV/PR8. Actb, β-actin. The percent density of total SFK (middle panel) and SFK phosphorylated at tyrosine 416 (right panel) against the densities of the corresponding proteins in IgG-treated, infected lungs after being normalized by the densities of β-actin. **, p<0.01. (**B**) Survival rate (%, upper panels) and body weight loss (%, lower panels) of WT mice intraperitoneally administrated with control IgG (left panels) and 38–2 mAb (right panel) together with 20 mg/mouse of DS 1 day before intranasal infection with 200 IFU of IAV/PR8. Error bars, SD. (**C**) Western blotting (upper panel) for total SFK, phosphorylated SFK (Tyr416), the cleaved caspase 3, and the viral protein M2 in the lungs from control IgG/DMSO-, 38–2 mAb/DMSO-, control IgG/DS-, and 38–2 mAb/DS-treated mice at 3 dpi with 200 IFU of IAV/PR8. Actb, β-actin. Lungs from uninfected, saline/DMSO-treated mice were used as a control. The percent density (lower panels) of total SFK, phosphorylated SFK (Tyr416), the cleaved caspase 3, and the viral protein M2 against the densities of the corresponding proteins in uninfected, saline/DMSO-treated lungs after being normalized by the densities of β-actin. *, p<0.05; **, p<0.01.

It has been reported that certain anti-PrP mAbs could activate SFK in neuronal and non-neuronal cells [19–22]. Therefore, to investigate the mechanism by which 38–2 mAb could provoke protection against lethal IAV infection, we first examined if SFK could be activated by 38–2 mAb in IAV-infected lungs, by performing Western blotting for SFK phosphorylation at tyrosine 416, the activated form of SFK, in the lungs of control IgG- and 38–2 mAb-treated WT mice at 3 dpi with IAV/PR8 (200 IFU). Phosphorylated SFK (Tyr416) was increased in 38–2 mAb-treated, IAV/PR8-infected lungs, compared to control lungs **(Fig 4A)**, indicating that 38–2 mAb could activate SFK in IAV-infected lungs. We then investigated the role of SFK activation in the 38–2 mAb-induced protective activity of PrP^C^ against lethal IAV infection, by administering 38–2 mAb and control IgG together with the SFK inhibitors dasatinib (DS) and PP2 into WT mice 1 day before intranasal infection with 200 IFU of IAV/PR8. DS and PP2 had no effects on the mortality of control IgG-treated, IAV/PR8-infected mice **(Fig 4B, S5 Fig, S6A Fig)**. However, both inhibitors abolished the protective activity of 38–2 mAb against lethal IAV infection **(Fig 4B, S5 Fig, S6A Fig)**. The survival rate of 38–2 mAb-treated, IAV/PR8-infected mice was markedly decreased by DS **(Fig 4B, S5 Fig)** and PP2 **(S6A Fig)**. The SFK activation and reduction of viral protein M2 and cleaved caspase 3 fragments in 38–2 mAb-treated, IAV/PR8-infected mice were also inhibited by DS **(Fig 4C)**. DS and PP2 inhibit not only SFKs but also other kinases such as c-Abl and c-Kit. We therefore similarly tested imatinib, which selectively inhibits c-Abl, platelet-derived growth factor receptor, and c-Kit [23]. Imatinib failed to inhibit the protective activity of 38–2 mAb against lethal IAV infection **(S6B Fig)**. These results indicate that 38–2 mAb could activate SFK in IAV-infected lungs, and that the SFK activation is essential for the 38–2 mAb-induced protective activity of PrP^C^ against lethal IAV infection.

### 38–2 mAb induces M2 macrophage polarization through activation of SFK

**Fig 5:**
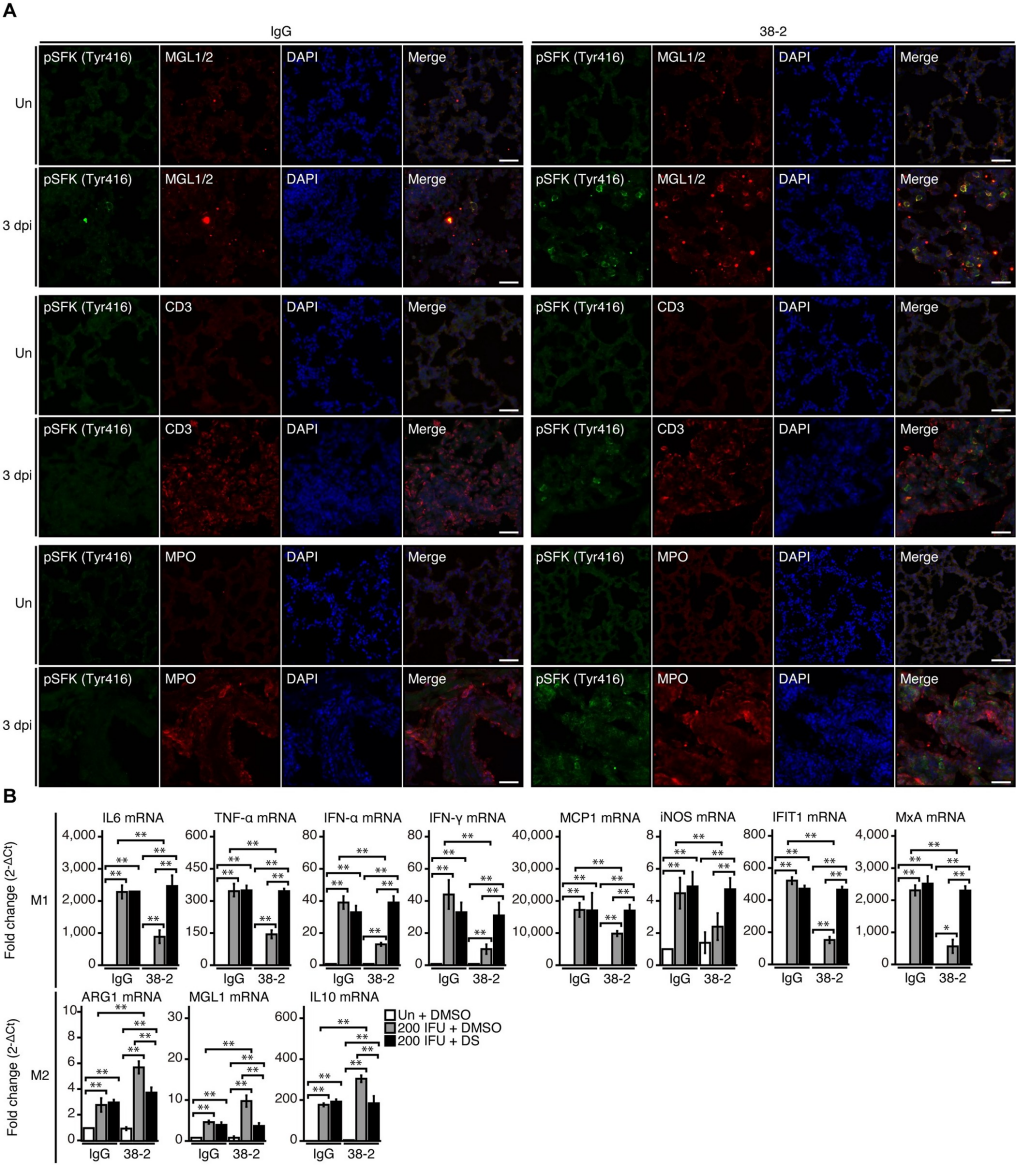
38–2 mAb activates SFK in M2 macrophage in IAV-infected lungs. (**A**) Double immunofluorescent staining for phosphorylated SFK (Tyr416) together with MGL1/2, CD3, and MPO in the lungs from control IgG- and 38–2 mAb-treated mice uninfected and at 3 dpi with 200 IFU of IAV/PR8. Un, uninfected. Bar, 200 **μ**m. (**B**) Fold mRNA expression (2^-ΔCt^) analyzed by real-time RT-PCR for IL-6, TNF-α, IFN-α, INF-γ, MCP-1, iNOS, IFIT1, MxA, ARG1, MGL1, and IL-10 in the lungs from control IgG/DMSO, control IgG/DS-, 38–2 mAb/DMSO-, and 38–2 mAb/DS-treated mice uninfected and at 3 dpi with 200 IFU of IAV/PR8 (n = 3 in each group). Un, uninfected.*, p<0.05;**, p<0.01.

To further elucidate the mechanism for the 38–2 mAb-induced protective activity of PrP^C^ against lethal IAV infection, we identified cell-types in which SFK is activated by 38–2 mAb in IAV/PR8-infected lungs. We used double immunofluorescent staining for phosphorylated SFK (Tyr416) and various cell markers including macrophage galactose-type C-type lectins 1 and 2 (MGL1/2) for macrophages, CD3 for T lymphocytes, and myeloperoxidase (MPO) for neutrophils. Signals for phosphorylated SFK (Tyr416) were increased in 38–2 mAb-treated, IAV/PR8-infected lungs compared to control lungs, and predominantly co-stained with MGL1/2, but not with CD3 or MPO **(Fig 5A)**, suggesting that 38–2 mAb could activate SFK in macrophages in IAV-infected lungs.

MGL1/2 is a specific marker for M2 macrophages. We therefore evaluated M1/M2 macrophage status in 38–2 mAb-treated, IAV/PR8-infected lungs, by investigating expression levels of M1 and M2 macrophage-specific genes using real-time reverse transcriptase-polymerase chain reaction (RT-PCR). Expression levels of the M1-specific genes such as those for IL-6, TNF-α, IFN-α, IFN-γ, monocyte chemotactic protein-1 (MCP-1) and inducible nitric oxide synthetase (iNOS) were lower in 38–2 mAb-treated, IAV/PR8-infected lungs than in control lungs **(Fig 5B)**. Consistently, the IFN-stimulated genes such as those for IFN-induced protein with tetratricopeptide repeats 1 (IFIT1) and Myxovirus resistance protein A (MxA) also had reduced expression in 38–2 mAb-treated, IAV/PR8-infected lungs, compared to control lungs **(Fig 5B)**. In contrast, the M2-specific genes including those for arginase 1 (ARG1), MGL1, and IL-10 were upregulated in 38–2 mAb-treated, IAV/PR8-infected lungs **(Fig 5B)**. In addition, DS increased expression of the M1-specific genes and decreased expression of the M2-specific genes in 38–2 mAb-treated, IAV/PR8-infected lungs **(Fig 5B)**. These results indicate that the 38–2 mAb-induced activation of SFK in macrophages could lead to predominance of M2 macrophages over M1 macrophages in IAV-infected lungs.

**Fig 6:**
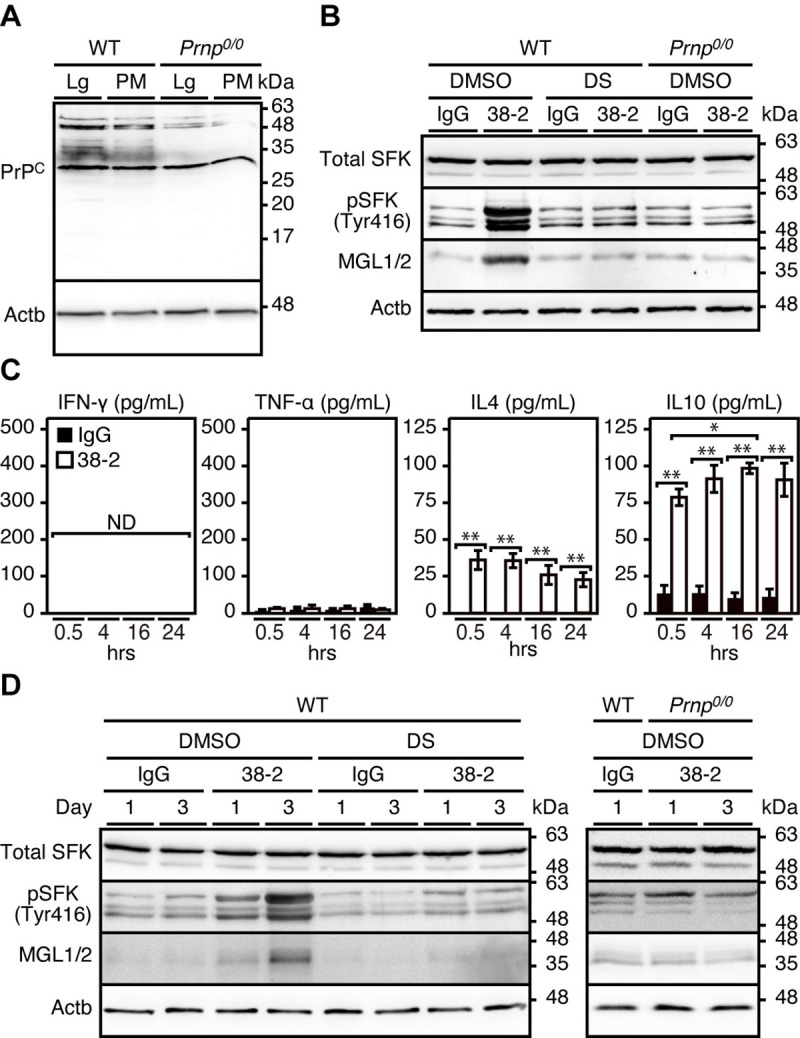
38–2 mAb induces M2 polarization through activation of SFK in peritoneal macrophages. (**A**) Uncropped, full picture of Western blotting for PrP^C^ with 38–2 mAb in the lungs (Lg) and peritoneal macrophages (PM) from WT and *Prnp*^*0/0*^ mice. Actb, β-actin. (**B**) Western blotting for total SFK, phosphorylated SFK (Tyr416), and MGL1/2 in peritoneal macrophages from WT and *Prnp*^*0/0*^ mice 3 hrs after treatment with control IgG and 38–2 mAb together with DS or control DMSO. (**C**) ELISA for INF-γ, TNF-α, IL-4, and IL-10 in the culture medium from WT peritoneal macrophages at 0.5, 4, 16, and 24 hours (hrs) after treatment with control IgG and 38–2 mAb.*, p<0.05;** p<0.01. (**D**) Western blotting for total SFK, phosphorylated SFK (Tyr416), and MGL1/2 in peritoneal macrophages (PM) from WT and *Prnp*^*0/0*^ mice 1 and 3 days after peritoneal injection with control IgG and 38–2 mAb together with control DMSO and DS.

To further clarify the role of 38–2 mAb-induced activation of SFK in macrophage polarization, we collected peritoneal macrophages and incubated them with 38–2 mAb. We first confirmed that peritoneal macrophages express PrP^C^ on Western blotting with 38–2 mAb **(Fig 6A)**. PrP^C^ is a glycoprotein with two glycosylation sites, therefore di-, mono-, and un-glycosylated forms of PrP^C^ are detected as several distinct bands **(Fig 6A)**. 38–2 mAb strongly cross-reacted with a non-PrP molecule of around 28 kDa in WT and *Prnp*^*0/0*^ peritoneal macrophages **(Fig 6A)**. Incubation with 38–2 mAb increased phosphorylated SFK (Tyr416) and MGL1/2 in WT macrophages, but not in *Prnp*^*0/0*^ macrophages **(Fig 6B)**. The SFK activation and MGL1/2 upregulation in 38–2 mAb-treated macrophages were suppressed by DS **(Fig 6B)**. The M2 cytokines, IL-4 and IL-10, but not the M1 cytokines, IFN-γ and TNF-α, were increasingly released into the medium from 38–2 mAb-treated WT macrophages **(Fig 6C)**. We also directly injected 38–2 mAb and control IgG into the peritoneal cavity of WT mice (1 mg/mouse) and collected their peritoneal macrophages 1 and 3 days after injection. Western blotting showed that WT peritoneal macrophages, but not *Prnp*^*0/0*^ peritoneal macrophages, had increased phosphorylated SFK (Tyr416) and MGL1/2 after treatment with 38–2 mAb **(Fig 6D)**. The increase in phosphorylated SFK (Tyr416) and MGL1/2 in 38–2 mAb-treated WT mice was abolished by DS **(Fig 6D)**. We also treated alveolar macrophages collected from the bronchoalveolar lavage fluids (BALF) of WT mice with 38–2 mAb, and investigated for expression of the M1- and M2-specific genes using real-time RT-PCR. The gene expression of M2-specific molecules, such as MGL1 and IL-10, was increased, but the expression of the M1-specific genes including those for TNF-α and IFN-γ was decreased, in 38–2 mAb-treated alveolar macrophages compared to control IgG-treated alveolar macrophages **(S7 Fig)**. These results suggest that interaction of 38–2 mAb with PrP^C^ on the surface of macrophages could lead to activation of SFK in the macrophages and polarize them to M2 macrophages.

To investigate which types of SFKs could be activated in macrophages treated with 38–2 mAb, we treated peritoneal macrophages with control IgG and 38–2 mAb, and subjected the cell lysates to immunoprecipitation with anti-phosphorylated SFK (Tyr416) Ab and then the precipitates to mass spectrometry. The anti-phosphorylated SFK (Tyr416) Ab most abundantly precipitated Lyn, with the abundance much higher in 38–2 mAb-treated peritoneal macrophages than in control peritoneal macrophages **(S8 Fig)**. To a lesser extent, Hck was also immunoprecipitated with higher abundance in 38–2 mAb-treated peritoneal macrophages than control peritoneal macrophages **(S8 Fig)**. Other SFKs including Fyn, Frk, Yes, Fgr, Lck, and Src were only marginally detectable in the precipitate **(S8 Fig)**. These results indicate that Lyn and to a lesser extent Hck could be predominantly phosphorylated in peritoneal macrophages treated with 38–2 mAb.

### 38–2 mAb is also therapeutically effective against lethal IAV infection

**Fig 7:**
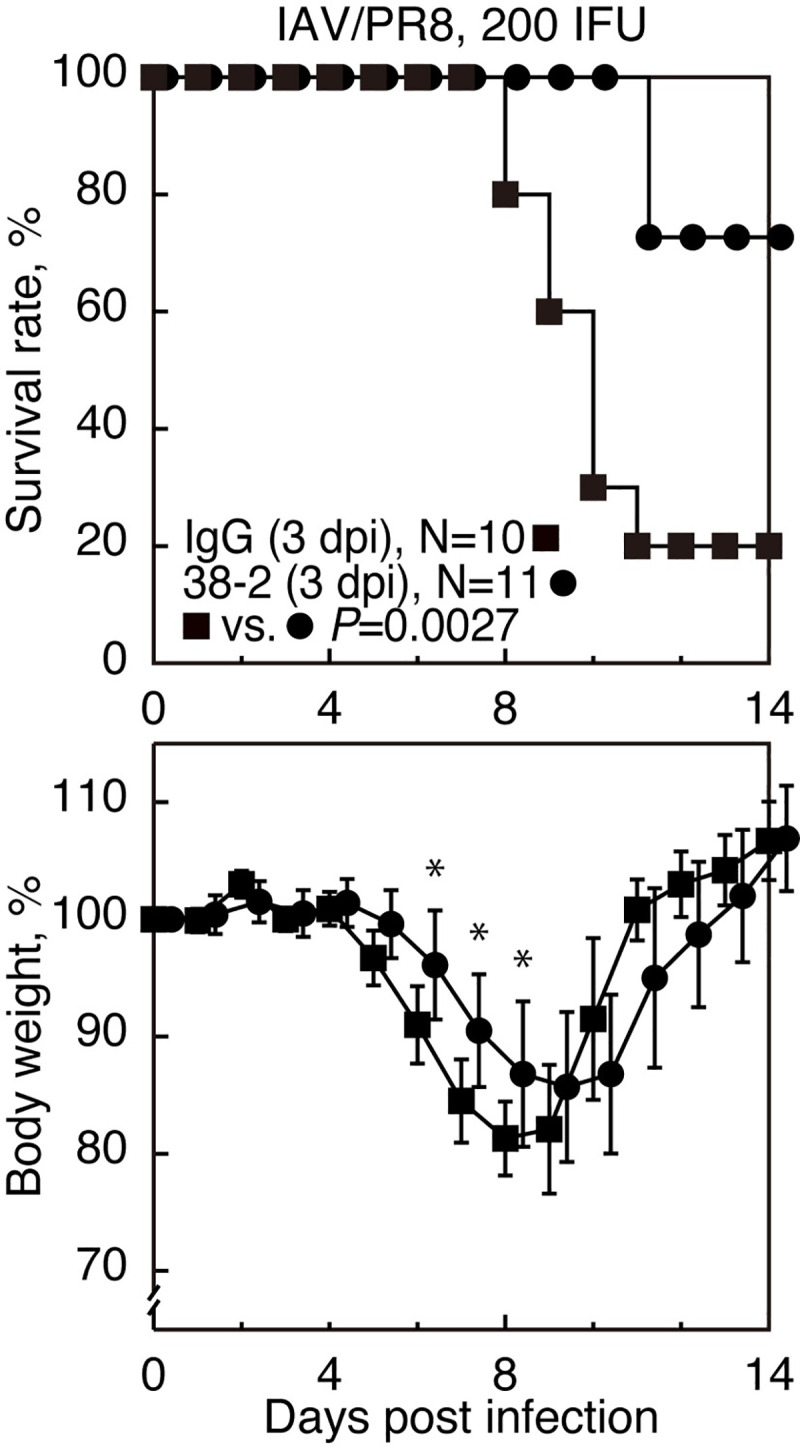
Therapeutic effects of 38–2 mAb against IAV infection. The survival rate (%, upper panel) and body weight loss (%, lower panel) of WT mice intraperitoneally administered with 38–2 mAb 3 days after intranasal infection with 200 IFU of IAV/PR8. Control IgG was similarly injected into WT mice 3 days after infection with 200 IFU of IAV/PR8. Error bars, SD. *, p<0.05.

We also investigated if 38–2 mAb could be therapeutically effective against lethal IAV infection, by intraperitoneally administering 38–2 mAb into WT mice (1 mg/mouse) 3 or 5 dpi with 200 IFU of IVA/PR8. Control IgG was similarly injected into WT mice at 3 dpi. Only 20% of control WT mice survived the infection **(Fig 7)**. However, the 3 dpi-treatment with 38–2 mAb significantly reduced the mortality of infected mice **(Fig 7)**. Weight loss was also reduced in 38–2 mAb-treated mice compared to control mice **(Fig 7)**. The treatment starting even at 5 dpi also tended to be slightly but not significantly effective at reducing the mortality of infected mice **(S9 Fig)**. These results indicate that 38–2 mAb could also be therapeutically effective against lethal IAV infection.

### 38–2 mAb also protects against lethal infection with other IAV strains

**Fig 8:**
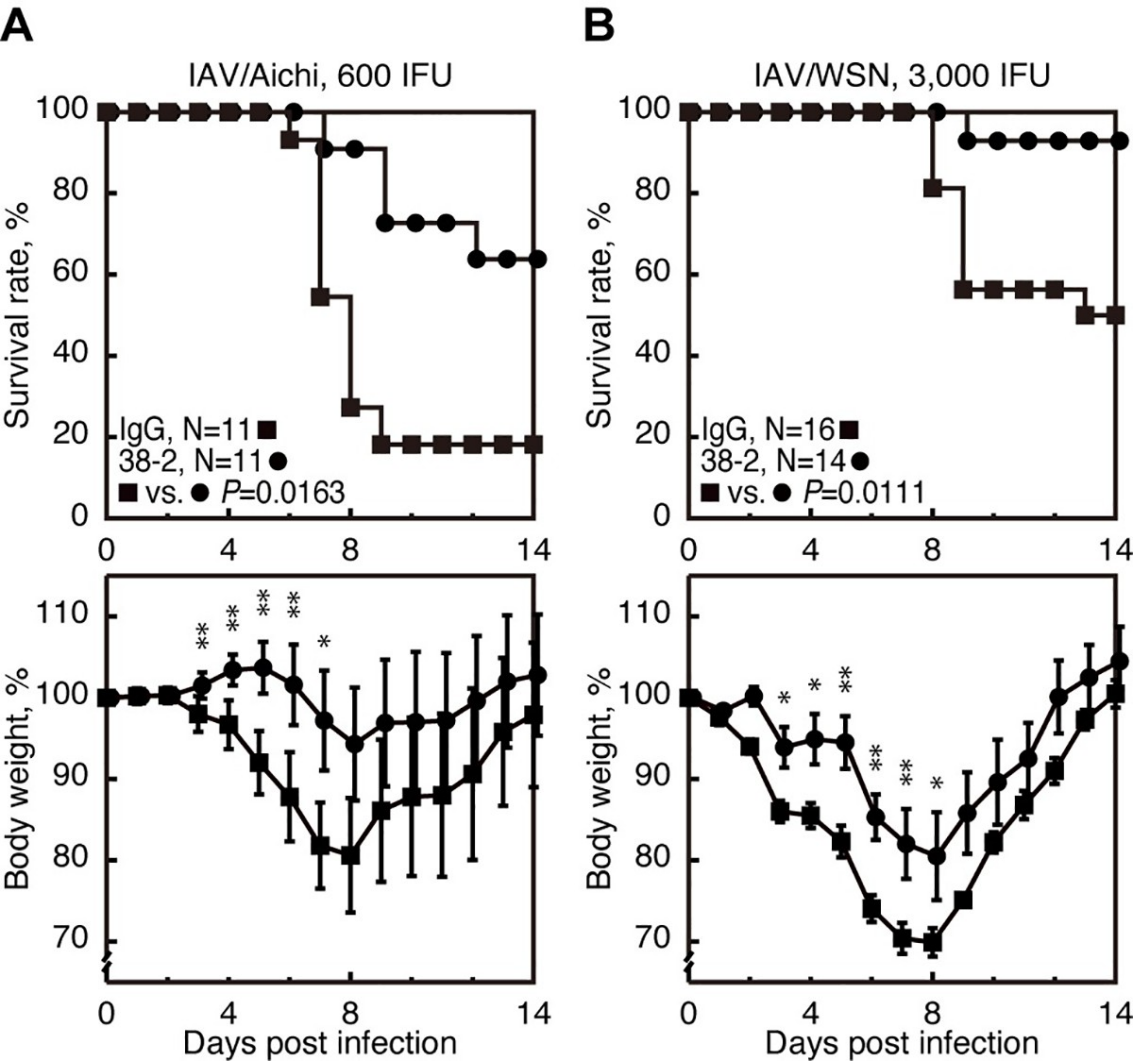
Protective activity of 38–2 mAb against other IAV strains. The survival rate (%, upper panels) and body weight loss (%, lower panels) of WT mice intraperitoneally administered with control IgG and 38–2 mAb 1 day before intranasal infection with 600 IFU of IAV/Aichi (**A**) and 3,000 IFU of IAV/WSN (**B**). Error bars, SD. *, p<0.05; **, p<0.01.

We further investigated if 38–2 mAb could protect against other IAV strains, by intraperitoneally injecting 38–2 mAb (1 mg/mouse) into WT mice 1 day before infection with 600 IFU of A/Aichi/2/68 (H3N2) (referred to as IAV/Aichi) and 3,000 IFU of A/WSN/33 (H1N1) (referred to as IAV/WSN). Control IgG was similarly injected into WT mice. Mortality of the control mice infected with IAV/Aichi and IAV/WSN was about 80% and 50%, respectively **(Fig 8A and 8B)**. However, 38–2 mAb significantly reduced the mortality to about 30% and 5% in mice infected with IAV/Aichi and IAV/WSN, respectively **(Fig 8A and 8B)**. Weight loss was also reduced in infected mice by 38–2 mAb **(Fig 8A and 8B)**. These results indicate that 38–2 mAb could confer protection against lethal infection with various IAV strains.

### Other anti-PrP mAbs also evoke protection against IAV infection through a similar mechanism

**Fig 9:**
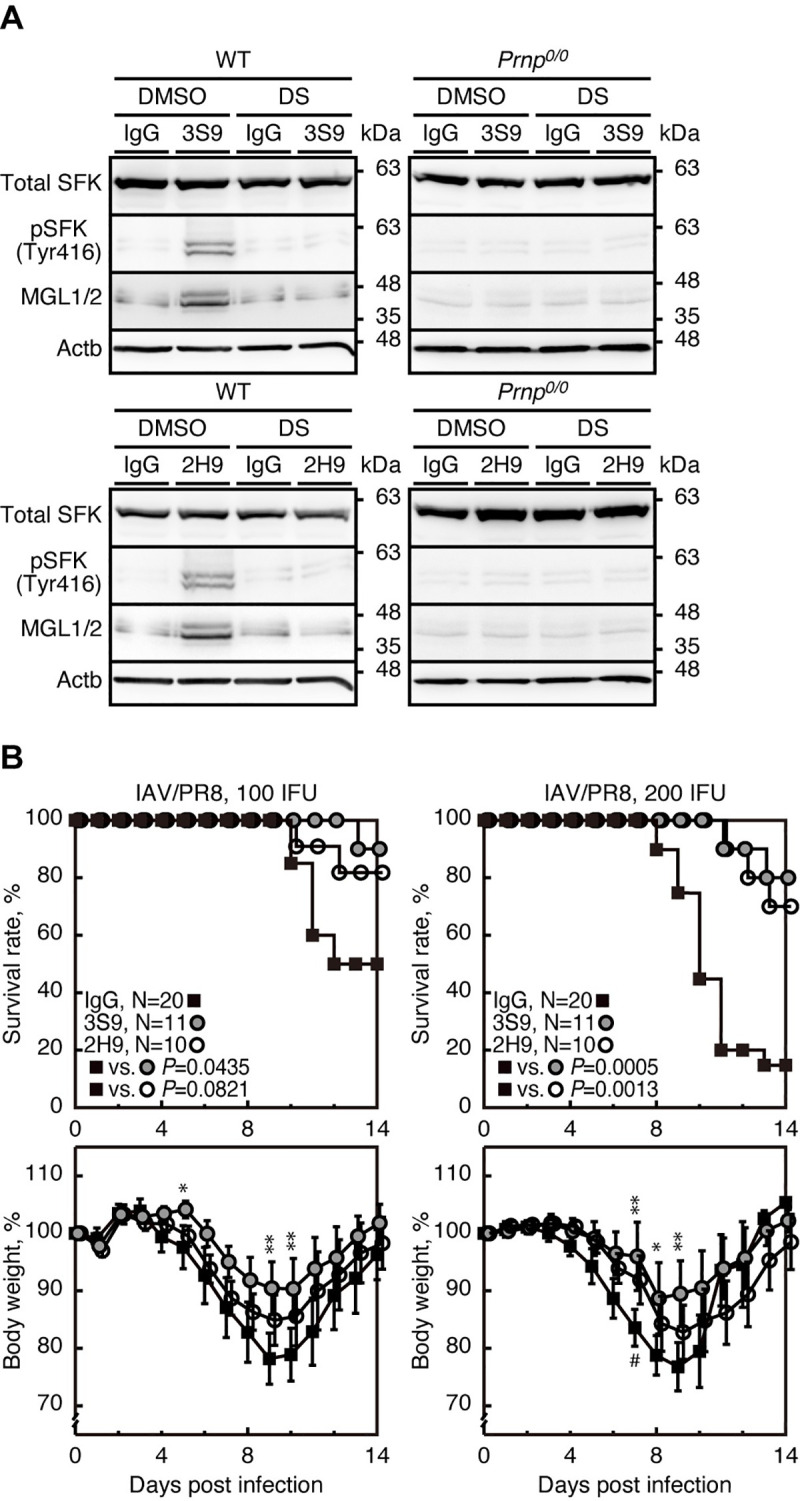
3S9 and 2H9 anti-PrP mAbs evokes protection against IAV infection through a similar mechanism. (**A**) Western blotting for total SFK, phosphorylated SFK (Tyr416), and MGL1/2 in peritoneal macrophages from WT and *Prnp*^*0/0*^ mice 3 hrs after treatment with control IgG and 3S9 and 2H9 mAbs together with control DMSO and DS. (**B**) Survival rate (%, upper panels) and body weight loss (%, lower panels) of WT mice intraperitoneally administered with control IgG and 3S9 and 2H9 mAbs 1 day before intranasal infection with 100 (left panels) or 200 IFU (right panels) of IAV/PR8. Error bars, SD. *, p<0.05; **, p<0.01; IgG vs 3S9. #, <0.05; IgG vs 2H9.

We also investigated if other anti-PrP mAbs could also evoke protective activity against lethal IAV infection through the same mechanism as 38–2 mAb. 3S9 and 2H9 anti-PrP IgG mAbs recognize residues 141–161 of mouse PrP^C^ and a discontinuous epitope included in residues 151–221, respectively [24,25]. 3S9 and 2H9 mAbs also activated SFK and upregulated MGL1/2 in peritoneal macrophages from WT mice but not from *Prnp*^*0/0*^ mice **(Fig 9A)**. The SFK activation and MGL1/2 upregulation in 3S9 or 2H9 mAb-treated WT macrophages were abolished by DS **(Fig 9A)**. These results indicate that, similarly to 38–2 mAb, 3S9 and 2H9 mAbs could activate SFK in macrophages and thereby induce M2 macrophage polarization. We then intraperitoneally administered 3S9 and 2H9 mAbs into mice (1 mg/mouse) 1 day before intranasal infection with 100 or 200 IFU of IAV/PR8. Both mAbs significantly reduced the mortality of IAV/PR8-infected mice **(Fig 9B)**. These results indicate that 3S9 and 2H9 mAbs could also evoke protective activity against lethal IAV infection by inducing M2 macrophage polarization through activating SFK.

### Anti-PrP mAbs specifically recognize PrP^C^ on the cell surface of macrophages

**Fig 10:**
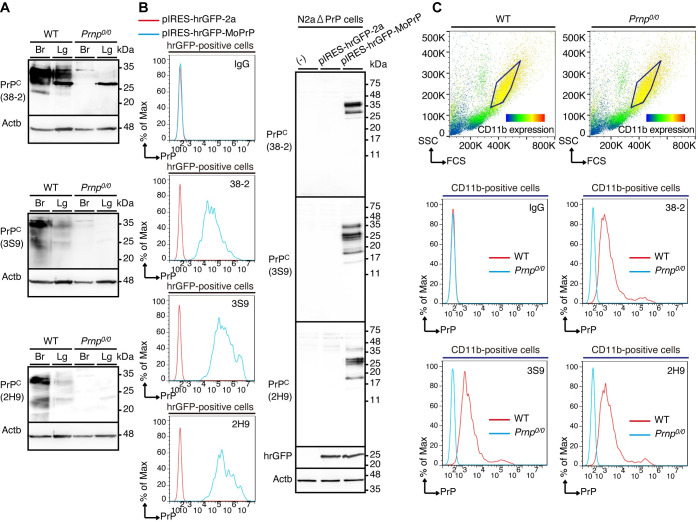
Anti-PrP mAbs specifically recognize PrP^C^ on the cell surface of macrophages. **(A**) Western blotting for PrP^C^ in the brains (Br) and lungs (Lg) from WT and *Prnp*^*0/0*^ mice with 38–2, 3S9, and 2H9 mAbs. (**B**) FACS analysis (left panel) with control IgG and 38–2, 3S9, and 2H9 mAbs of hrGFP-positive N2aΔPrP cells 3 days after transfection with control vector and pIRES-hrGFP-MoPrP vector. Western blotting (right panel) with 38–2, 3S9, and 2H9 mAbs of N2aΔPrP cells transfected with control vector and pIRES-hrGFP-MoPrP vector. (**C**) FACS analysis with control IgG and 38–2, 3S9, and 2H9 mAbs of CD11b-positive peritoneal macrophages from WT and *Prnp*^*0/0*^ mice.

To investigate whether 38–2, 3S9, and 2H9 mAbs could specifically recognize PrP^C^ expressed on the cell surface of macrophages, we first confirmed that all of these mAbs detected PrP^C^ in the brains and lungs of WT mice by Western blotting **(Fig 10A, S10A–S10C Fig)**. Consistent with PrP^C^ being expressed as different glycosylated forms, several distinct bands corresponding to the different glycosylated forms of PrP^C^ were detected with these mAbs in WT brains and lungs, but not in *Prnp*^*0/0*^ brains **(Fig 10A, S10A–S10C Fig)**. PrP^C^ was detected more easily with 38–2 mAb than with 3S9 and 2H9 mAbs, and the band patterns of PrP^C^ were different with each of the mAbs **(Fig 10A, S10A–S10C Fig)**. This could be because these mAbs recognize different epitopes. These mAbs also weakly cross-reacted with several other molecules except for a molecule of around 28 kDa that was strongly cross-reacted with 38–2 mAb in WT and *Prnp*^*0/0*^ lungs **(Fig 10A, S10A–S10C Fig)**. A similar cross-reactive band was detected with 38–2 mAb in peritoneal macrophages **(Fig 6A)**. It is thus possible that the cross-reactive band around 28 kDa in WT and *Prnp*^*0/0*^ lungs might be derived from alveolar macrophages.

We then performed FACS analysis with these mAbs. To avoid confusion that might be caused by expression of endogenous PrP^C^, we used PrP^C^-knockout cells, N2aΔPrP cells [26]. The cells were transfected with control vector or pIRES-hrGFP-MoPrP vector, which encodes mouse PrP^C^ together with the transfection marker humanized Renilla green fluorescent protein (hrGFP) under the control of an internal ribosomal entry site (IRES) motif. Western blotting with 38–2, 3S9, and 2H9 mAbs showed several distinct bands of PrP^C^ in pIRES-hrGFP-MoPrP-transfected cells, but not in control vector-transfected cells **(Fig 10B)**. We analyzed hrGFP-positive cells for PrP^C^ expression with these mAbs by FACS. Compared to control IgG, these mAbs showed a shifted signal in pIRES-hrGFP-MoPrP-transfected cells, but not in control vector-transfected cells **(Fig 10B)**, indicating that these mAbs could specifically recognize PrP^C^ expressed on the cell surface of N2aΔPrP cells. We also performed FACS analysis of CD11b-positive peritoneal macrophages from WT and *Prnp*^*0/0*^ mice for PrP^C^ expression with 38–2, 3S9, and 2H9 mAbs. These mAbs also showed a shifted signal in CD11b-positive WT macrophages, but not in CD11b-positive *Prnp*^*0/0*^ macrophages **(Fig 10C)**, indicating that these mAbs could also specifically recognize endogenous PrP^C^ on the cell surface of peritoneal macrophages.

## Discussion

In the present study, we showed that targeting of PrP^C^ with three different anti-PrP mAbs examined, including 38–2, 3S9 and 2H9, conferred protection against lethal infection with IAVs in WT mice. These anti-PrP mAbs mitigated inflammation, inflammatory cytokine production, epithelial cell damage and virus production in IAV-infected lungs and eventually reducing the morbidity and mortality of IAV-infected mice. 38–2 mAb failed to protect *Prnp*^*0/0*^ mice from lethal infection with IAV/PR8, confirming that targeting of PrP^C^ with anti-PrP mAbs could elicit the protective activity against IAV infection. We previously showed that, although *Prnp*^*0/0*^ mice were highly susceptible to IAV infection, they were successfully rescued from lethal infection with the same IFU of IAV/PR8 as used in WT mice, by treatment with butylated hydroxyanisole, a radical oxygen species (ROS) scavenger, and allopurinol, the inhibitor of xanthine oxidase, which is a major ROS-generating enzyme in IAV-infected lungs [3]. These results indicate that the unsuccessful protection of *Prnp*^*0/0*^ mice by 38–2 mAb could not be due to the higher sensitivity of *Prnp*^*0/0*^ mice to IAV infection, but due to lack of PrP^C^ in *Prnp*^*0/0*^ mice. It is thus conceivable that PrP^C^ could be a new therapeutic target for IAV infection.

It has been previously reported that PrP^C^ could regulate SOD1 activity, thereby reducing oxidative stress in IAV-infected lungs and providing protection against IAV infection in mice [3]. However, 38–2 mAb did not increase SOD1 activity in IAV-infected lungs, indicating that the SOD1-mediated anti-oxidative activity of PrP^C^ is not involved in the anti-PrP mAb-induced protective activity of PrP^C^ against IAV infection. We showed that Fab fragments of 38–2 mAb reduced the mortality of IAV-infected mice, similarly to its parental 38–2 mAb. This suggests that the protective activity of the anti-PrP mAbs against IAV infection does not depend on cross-linking of PrP^C^ or the Fc-dependent Ab functions, such as Ab-dependent cell-mediated cytotoxicity, Ab-dependent cellular phagocytosis, and Ab-mediated complement activation leading to complement-dependent cytotoxicity. Consistent with other reports demonstrating that stimulation of PrP^C^ with certain anti-PrP mAbs activated SFK in cultured cells [19–22], we observed that SFK was activated in 38–2 mAb-treated, IAV-infected lungs. Moreover, DS and PP2, which inhibit SFKs as well as c-Abl and c-Kit kinases, but not the c-Abl and c-Kit kinase-specific inhibitor, imatinib [23], abolished the 38–2 mAb-induced protective activity of PrP^C^ against IAV infection, decreasing the survival rate of 38–2 mAb-treated, IAV-infected mice, suggesting that the anti-PrP mAb-induced SFK activation is essential for the anti-PrP mAb-induced protective activity of PrP^C^ against IAV infection. Administration with DS alone did not affect the mortality of IAV/PR8-infected mice, suggesting that SFK is not critically involved in the pathogenesis of IAV infection. Consistent with this, phosphorylated SFK (Tyr416) was not increased in the lungs of control IgG-treated, IAV/PR8-infected mice.

We found that SFK was predominantly activated in M2 macrophages in 38–2 mAb-treated, IAV-infected lungs, and that DS reduced M2 macrophages and conversely increased M1 macrophages in 38–2 mAb-treated, IAV-infected lungs. We also found that 38–2, 3S9, and 2H9 mAbs activated SFK in peritoneal macrophages from WT mice, but not from *Prnp*^*0/0*^ mice, and induced M2 polarization in WT macrophages *in vitro*, and that DS inhibited the anti-PrP mAbs-induced M2 polarization in WT macrophages. We further showed that 38–2 mAb activated SFK in peritoneal macrophages and induced M2 macrophage polarization *in vivo*. FACS analysis of WT and *Prnp*^*0/0*^ peritoneal macrophages revealed that 38–2, 3S9, and 2H9 mAbs specifically recognized PrP^C^ on the cell surface of WT peritoneal macrophages. These results suggest that anti-PrP mAbs could activate SFK through interaction with PrP^C^ on the cell surface of macrophages and induce M2 macrophage polarization. Phosphorylated Lyn and to a lesser extent phosphorylated Hck were predominantly precipitated with anti-phosphorylated SFK (Tyr416) Ab in 38–2 mAb-treated peritoneal macrophages, suggesting that these SFKs might be involved in the anti-PrP mAb-induced M2 polarization in macrophages. These results also might be consistent with Lyn and Hck being highly expressed in macrophages [27]. IL-4 and IL-10, both of which are known to induce M2 macrophage polarization through the STAT6 pathway [28,29], were abundantly secreted into the culture medium of peritoneal macrophages after incubation with 38–2 mAb. It is thus possible that IL-4 and IL-10 could be involved in the anti-PrP mAbs-induced M2 macrophage polarization.

Macrophages are phagocytic cells that play an important role in infectious diseases by triggering innate immunity and regulating various stages of inflammation [28,29]. Based on their phenotype and function, macrophages can be grouped into two subclasses: pro-inflammatory M1 and anti-inflammatory M2 macrophages [28,29]. In the early stages of infection, macrophages are polarized to a M1 phenotype. They produce inflammatory cytokines including IL-1, IL-6, and TNF-α, trigger innate immunity, and eventually protect the host from pathogen colonization. In contrast, M2 macrophages are generated in the late stages of infection, terminating the inflammation and repairing damaged tissues, by releasing anti-inflammatory cytokines such as tumor growth factor-β (TGF-β) and IL-10 and removing damaged cells [28,29]. Several reports indicate that M2 macrophages could be therapeutically beneficial for IAV infection. Mice intranasally administered with exogenous M2 macrophages or transgenically expressing granulocyte-macrophage colony-stimulating factor in lung epithelial cells to increase M2 macrophages were shown to display better clinical scores than control mice after infection with IAV [30,31]. It is thus likely that the anti-PrP mAb-induced M2 macrophage generation through SFK activation is the underlying mechanism for the anti-PrP mAb-induced protective activity of PrP^C^ against IAV infection.

Neuronal loss was reported in mice stereotaxically injected into the hippocampus with D13 and P anti-PrP mAbs, both of which recognize epitopes within residues 95–150 of PrP^C^ [32]. Sonati *et al*. also reported that anti-PrP mAbs, which recognize the C-terminal globular domain of PrP^C^, induced degeneration of cerebellar granule cells in cerebellar organotypic cultured slices and in mice by causing oxidative stress, which was mainly produced by NADPH oxidase 2 [33]. These results have raised a safety concern for therapeutic application of PrP^C^-targeting agents, including anti-PrP mAbs. However, other investigators reported no neuronal loss in the hippocampus of mice injected with not only D13 and P anti-PrP mAbs but also two other different anti-PrP mAbs [34]. We also observed that treatment with 38–2 mAb suppressed the caspase 3 activity in IAV-infected lungs, indicating that 38–2 mAb is not toxic to cells. Further studies are necessary to clarify whether PrP^C^-targeting agents including anti-PrP mAbs are safe for therapeutic application.

38–2 and 3S9 mAbs recognize residues 41–45 and 141–161 of PrP^C^, respectively, and 2H9 mAb reacts with a discontinuous epitope included in residues 151–221 of PrP^C^ [24,25]. Other anti-PrP mAbs, including SAF32, SAF61, and 3F4, which recognize residues 86–103, 142–160, and 109–112 of PrP^C^, respectively, were also shown to induce SFK activation in cultured cells [19–22]. It is thus likely that various sites, not just a single site, in PrP^C^ could induce SFK activation after targeting with anti-PrP mAbs. The mechanism by which the binding of anti-PrP mAbs to extracellular GPI-anchored PrP^C^ leads to activation of intracellular SFK still remains challenging. It has been reported that the anti-PrP mAbs-mediated activation of SFK in neuronal cells is dependent on caveolin-1 [19]. However, caveolin-1 is an integrated plasma membrane protein forming the cytoplasmic ridges of caveola, with both the N- and C-termini facing the cytoplasm [35]. It is therefore unlikely that caveolin-1 could directly transduce the signal from the GPI-anchored extracellular PrP^C^ to the intracellular SFK after stimulation with anti-PrP mAbs. It has been shown that a small proportion of PrP^C^ molecules on the plasma membrane could adopt transmembrane forms, with either the N- or C-terminus being exposed to the cytoplasm [36,37], suggesting that the transmembrane forms of PrP^C^ could directly interact with and activate SFK in the cytoplasm after stimulation with anti-PrP mAbs. However, 38–2 mAb reacts with the N-terminal residues of PrP^C^ and 3S9 and 2H9 mAbs recognize the C-terminal residues, indicating that the transmembrane forms of PrP^C^ are not responsible for the anti-PrP mAb-induced activation of SFK. Another possibility is that an as yet unidentified transmembrane molecule(s) could form a link between PrP^C^ and SFK. Anti-clathrin Ab was reported to partially reduce the anti-PrP mAb-induced activation of SFK [19], suggesting that clathrin might be a candidate molecule connecting PrP^C^ and SFK. Other candidates have been also suggested, including epidermal growth factor receptor and neural cell adhesion molecule [20,38]. It has been shown that Alzheimer amyloid-β oligomer could bind to PrP^C^ around the central residues 97–100 and activate SFK in neurons [39], suggesting that these residues might be involved in the anti-PrP mAb-induced activation of SFK. The polybasic region and the so-called octapeptide region in the N-terminal domain of PrP^C^ have been suggested to be important to induce intracellular signaling from PrP^C^ [33,40]. However, it is unknown whether or not these regions could be involved in the anti-PrP mAb-induced activation of SFK. Identification of a molecule(s) connecting PrP^C^ and SFK and mediating the anti-PrP mAb-induced activation of SFK and the site involved in the anti-PrP mAb-induced activation of SFK would be helpful for further understanding of not only the mechanism for the anti-PrP mAb-induced M2 macrophage polarization, but also the role of the anti-PrP mAb-induced SFK activation in the normal function of PrP^C^ in neuronal cells.

In short, we showed that targeting of PrP^C^ with anti-PrP mAbs could induce M2 macrophage polarization though activation of SFK and thereby confer protection against lethal influenza infection in mice. These results revealed a novel function of PrP^C^ in M2 macrophage polarization, and suggest that the anti-PrP mAb-induced M2 macrophage-polarizing activity of PrP^C^ could be a novel therapeutic target for influenza infection. Many lines of evidence are emerging that induction of M2 macrophages might be beneficial in other non-infectious chronic inflammatory diseases, including type 2 diabetes, atherosclerosis, and obesity [28,29,41]. Therefore, it might be interesting to investigate if treatment with anti-PrP mAbs could be therapeutically beneficial in these diseases. However, careful attention should be paid to the potential detrimental effects that might be caused by the forced polarization into M2 macrophages by PrP^C^-targeting agents including anti-PrP mAbs.

## Materials and methods

### Ethics statement

The Ethics Committee of Animal Care and Experimentation of Tokushima University approved this study (approval number T30-100 and T2019-81). Mice and chicken embryos were cared for in accordance with The Guiding Principle for Animal Care and Experimentation of Tokushima University and with Japanese Law for Animal Welfare and Care. 10-day old chicken embryos were purchased from Ishii Co., Ltd., Tokushima, Japan.

### Animals

C57BL/6 mice were purchased from Japan SLC Inc. (Shizuoka, Japan). *Prnp*^*0/0*^ mice (kindly provided by Dr. Stanley B. Prusiner) used in this study had been obtained elsewhere by at least more than 9 time-backcrosses to C57BL/6 with *Prnp*^*0/0*^ mice originally carrying a mixed background of C57BL/6×129Sv×FVB mice [42,43]. Mice were housed under specific pathogen-free conditions in cages of 5–6 animals with water and food *ad libitum*. Cages were provided with standard softwood bedding. Mice were kept on a standard 12:12 light:dark cycle. The mouse experiments carried out in this study were reviewed by the Ethics Committees of Animal Care and Experimentation of Tokushima University every year.

### Antibodies

Anti-podoplanin antibody (Cat. No. D190-3) and anti-β-actin antibody (Cat. No. M177-3) from MBL (Nagoya, Japan;), anti-SP-C (Cat. No. sc-7706) and anti-CC10 antibodies (Cat. No. sc-9772) from Santa Cruz Biotechnology (Santa Cruz, CA), anti-pro-caspase 3 (Cat. No. 9662S), anti-cleaved caspase 3 (Cat. No. 9664S), anti-total SFK (Cat. No. 2109S) and anti-phosphorylated SFK (Tyr416) antibodies (Cat. No. 2101S) from Cell Signaling (Beverly, MA), anti-PB1 (Cat. No. GTX125923), anti-NS1 (Cat. No. GTX125990), anti-M2 (Cat. No. GTX125951) and anti-NP (Cat. No. GTX125989) antibodies from GeneTex (Irvine, CA), anti-SOD1 antibody (Cat. No. ab13498) from Abcam (Cambridge, UK), anti-CD3 (Cat. No. MAB4841), anti-MGL1/2 (Cat. No. AF4297) and HRP -conjugated anti-goat IgG antibodies (Cat. No. HAF017) from R&D systems (Minneapolis, MN), anti-MPO antibody from Proteintech (Rosemont, IL; Cat. No. 66177-1-Ig), PE anti-mouse/human CD11b antibody (Cat. No. 101207) from Biolegend (San Diego, CA), vitality full length hrGFP polyclonal antibody (Cat. No. 240141) from Agilent (Santa Clara, CA), HRP-conjugated anti-mouse IgG antibodies (Cat. No. NA931), HRP-conjugated anti-rabbit IgG antibodies (Cat. No. NA934), HRP-conjugated anti-rat IgG antibodies (Cat. No. NA935) from GE Healthcare (Buckinghamshire, UK), Alexa Fluor 596 donkey anti-mouse IgG antibody (Cat. No. A21203), Alexa Fluor 488 goat anti-rabbit IgG antibody (Cat. No. A11008), Alexa Fluor 596 donkey anti-goat IgG antibody (Cat. No. A11058), Texas Red-X goat anti-rat IgG antibody (Cat. No. T6392), and Alexa Fluor 647 goat anti-mouse antibody (Cat. No. A28181) from Invitrogen (Carlsbad, CA).

### Virus preparation

IAV/PR8, IAV/Aichi, and IAV/WSN were grown in the allantoic sac of 11-day-old chicken embryos (Ishii Co., Ltd.) at 36°C for 48 hours. Eggs were chilled at 4°C for at least 4 hours prior to harvesting the allantoic fluids. Cellular debris was removed by centrifugation at 2,380×g at 4°C for 30 min. The clarified supernatant was layered over a 20% sucrose cushion and centrifuged at 25,000×g at 4°C for 120 min. The virus pellet was suspended in phosphate-buffered saline (PBS), and stored in multiple aliquots at -80°C until use.

### Intranasal infection of IAVs

After anesthesia with intramuscular injection of ketamine (Daiichi-Sankyo Propharma Co., LTD., Tokyo, Japan), male mice aged 5 weeks were intranasally inoculated with IAVs in a total volume of 20 **μ**L (10 **μ**L in each nasal cavity), and monitored for survival, weight loss, and clinical signs of illness for 14 days. The IAV stock aliquots were thawed and diluted in saline before use. To remove tissues, mice were anesthetized with ketamine (Daiichi-Sankyo Propharma Co., LTD.) and subjected to perfusion through the right ventricle with PBS.

### Virus titer determination

Virus titers were expressed as IFU/mL, which was determined using Madin-Darby canine kidney (MDCK) cells (kindly provided by Dr. Takaaki Nakaya, Kyoto Prefectural University of Medicine) as follows. MDCK monolayer cells were inoculated with a 10-fold serial dilution of each sample of interest for 14 hours at 37°C. The cells were then fixed with 4% paraformaldehyde (PFA), permeabilized with 0.3% Triton-X100 in PBS, and immunostained with anti-NP monoclonal antibodies (GeneTex). Signals were visualized using horseradish peroxidase (HRP)-conjugated anti-rabbit IgG antibodies (GE Healthcare) and TrueBlue Peroxidase Substrate (KPL, Gaithersburg, MD). IFU/mL was defined as the number of the cells positive for the anti-NP signals in 1 mL of each sample.

### Antibody treatments

Mice were intraperitoneally injected with the indicated amounts of respective antibodies at indicated times. Control mouse polyclonal IgG was purchased from Sigma-Aldrich (St Louis, MO).

### Purification of anti-PrP mAbs

38–2 [17], 3S9 [24,25], and 2H9 hybridoma cells [24,25] were inoculated into the peritoneal cavity of BALB/cSlc-nu/nu mice (Japan SLC Inc.) pretreated with pristane (Sigma). Abs in their ascites were then purified using a MAbTrap Kit (GE Healthcare) according to the manufacturers’ instructions.

### Purification of 38–2 Fab

38–2 Fab and control mouse IgG Fab fragments were prepared using a Mouse IgG_1_ Fab and F(ab’)_2_ Preparation Kit (Thermo Scientific Inc., Waltham, MA). In brief, 38–2 mAb and control mouse IgG (Sigma) were respectively subjected to proteolysis with immobilized ficin. The digested Fc portion and undigested IgG fragment were removed by passing over a column of immobilized protein A 3 times and the resulting elutes containing the Fab fragments was collected. The Fab concentration was determined at 280 nm using a Nanodrop 1000 spectrophotometer (Thermo Scientific Inc.).

### Oral administration with DS, PP2 and Imatinib

DS was purchased from Santa Cruz Biotechnology, PP2 from Tocris bioscience (Bristol UK), and Imatinib mesylate from Selleck Chemicals (Houston, TX). DS and PP2 were dissolved in dimethyl sulfoxide (DMSO) and stored at -20°C. Imatinib mesylate was dissolved in water and stored at -20°C until use. DS was orally administered at 10 mg/kg/day or 20 mg/kg/day, and PP2 was administered at 5 mg/kg/day using a sonde needle daily from -1 to 4 dpi. Imatinib mesylate was orally administered at 200 mg/kg/day twice a day from -1 to 14 dpi.

### Homogenization

Cells and tissues were homogenized in lysis buffer (0.5% Triton X-100, 0.5% Sodium deoxycholate, 150 mM NaCl, 50 mM Tris-HCl, pH 7.4, 1 mM EDTA) containing protease inhibitor cocktail (Nakalai Tesque, Kyoto, Japan) and phosphatase inhibitor cocktail (Nakalai Tesque). The homogenate was centrifuged at 1,000×g at 4°C. Protein concentration of the homogenates was measured using the BCA method (Thermo Scientific Inc.).

### Western blotting

Total proteins were denatured by boiling for 5 min in Laemmli’s sample buffer and subjected to sodium dodecyl sulfate-polyacrylamide gel electrophoresis. Proteins were transferred onto Immobilon-PVDF membranes (Millipore, Bedford, MA), and membranes were blocked for 2 hours with 5% non-fat dry milk-containing TBST (0.1% Tween-20, 100 mM NaCl, 10 mM Tris-HCl, ph 7.6). Primary antibodies were incubated with the membrane overnight at 4°C. Signals were visualized using horseradish peroxidase-conjugated anti-mouse, anti-rabbit, anti-goat, and anti-rat IgG antibodies (GE Healthcare), and detected using a chemiluminescence image analyzer LAS-4000 mini (Fujifilm Co., Tokyo, Japan). Signal intensities were measured using ImageJ 64 software (NIH, Bethesda, MD). The average and standard deviation (SD) of the signals were calculated using Microsoft EXCEL software, version 16.37.

### Pathological examinations

Lung tissues were fixed with 4% PFA, dehydrated, embedded in paraffin, and cut into 5 **μ**m-thick tissue sections. The sections were deparaffinized and stained with hematoxylin-eosin (H-E).

### Determination of atelectatic lung areas

The atelectatic lung area was evaluated using Photoshop software (Adobe, San Jose, CA) and ImageJ software (NIH, Bethesda, MD). Briefly, the original RGB color images of H-E stained lung sections were converted to black-on-white images by use of Photoshop software and saved in TIFF format. The binary images in TIFF format were again inverted into a white-on-black image using the ImageJ application. Atelectatic lung area was expressed as the area of white pixels, which represent the lung parenchyma, against total lung area (white and black pixels).

### Immunohistochemistry

Frozen 5 **μ**m-thick tissue sections were fixed in cold acetone for 15 min, air-dried, and treated for 30 min with a blocking reagent [10% fetal bovine serum (FBS) in PBS]. After washing with PBS, the sections were incubated with primary antibodies overnight at 4°C, and Alexa Fluor 488 goat anti-rabbit IgG (Invitrogen) for anti-NP and anti-pSFK (Tyr416) antibodies, Alexa Fluor 594 donkey anti-goat IgG (Invitrogen) for anti-MGL1/2 antibody, Texas Red-X goat anti-rat IgG (Invitrogen) for anti-CD3 antibody, and Alexa Fluor 594 donkey anti-rabbit IgG (Invitrogen) for anti-MPO antibody for 2 hrs at room temperature (RT). The sections were mounted with CC/Mount (Diagnostic BioSystems, Pleasanton, CA) containing DAPI (Dojindo Laboratories, Kumamoto, Japan). Fluorescent images were visualized using BIOREVO BZ-9000 (Keyence, Osaka, Japan).

### Evaluation of NP-positive cells in the airway and parenchymal areas

Lung slices (n = 3 slices × 3 mice in each group) were stained with anti-NP antibody (GeneTex). The NP-positive cells in large airway (larger than 100 **μ**m diameter) was evaluated by scoring 0 to 5 as followed: Score 0, intact epithelial cells in the airway without NP-positive cells; score 1, intact epithelial cells in the airway with <50% NP-positive cells; score 2, intact epithelial cells in the airway with >50% NP-positive cells; score 4, <50% epithelial cell injuries in the airway with NP-positive cells; score 5, >50% epithelial cell injuries in the airway with NP-positive cells. The NP positive cells in the parenchymal areas were counted using Photoshop software (Adobe) and ImageJ particle count command (NIH).

### ELISA for cytokines

IL-6, IFN-γ, TNF-α, IL-4, and IL-10 levels in samples were determined using a Quantikine ELISA kit (R&D systems, Minneapolis, MN) according to the respective protocols provided by the manufacturer. In brief, the samples were diluted 1:1 with the assay diluent provided in the kit and added to the ELISA microplate wells. The plates were then left for 2 hours at RT. The wells were washed with the wash buffer 5 times and added with the mouse IFN-γ, TNF-α, IL-4, and IL-10 conjugate for 2 hrs. The wells were then washed with the wash buffer and the substrate reagent was added for 30 min. The reaction was stopped by addition of the stop solution. The optical density of each well was measured at 450 nm in an automated microplate reader (Thermo LabSystems, Waltham, MA).

### TUNEL staining

TUNEL staining was performed using the *in situ* cell death detection kit and fluorescein (Roche Diagnostics, Mannheim, Germany) in accordance with the manufacturers’ protocol. In brief, deparaffinized tissue sections were treated with 20 **μ**g/mL proteinase K (Wako Pure Chemical Industries) in 10 mM Tris-HCl for 30 min at RT and incubated in the TUNEL reaction mixture for 1 hr at 37°C in a humidified dark chamber. The sections were washed with PBS for 5 min 3 times and signals were detected using BIOREVO BZ-9000 (Keyence).

### RNA extraction and real-time RT-PCR

Total RNA was first extracted from cells and tissues using RNeasy Mini Kit (QIAGEN, Valencia, CA). Homogenates in buffer RLT were transferred to a QIAshredder spin column (QIAGEN). The flow-through was mixed with 1 volume of 70% ethanol and then transferred to an RNeasy spin column (QIAGEN). Total RNA bound to the membrane was washed with buffer RW1 and then with buffer RPE, and eluted with RNase-free water. Total RNA was used in a reverse transcription reaction using M-MLV Reverse Transcriptase (Promega, Madison, WI) and real-time PCR was performed with FastStart Universal SYBR green PCR Master (Roche Diagnostics) according to the manufacturers’ protocols. Optimal PCR conditions were 40 cycles of 3-step cycling (Denaturation at 95°C for 15 sec, Annealing at 56°C for 30 sec, Extension at 72°C for 50 sec) after an initial denaturation step (95°C for 10 min). For the relative comparison of each gene expression, the data of real-time PCR were analyzed with 2^-ΔCt^ method. All real-time PCRs were run using the Applied Biosystems 7300 system (Foster City, CA), and the cycle threshold (CT) value for each sample was obtained using ABI 7300 system sequence detection software, version 1.3. Relative quantitative levels for each sample were calculated on the basis of the CT value by standard curve methods and were normalized to endogenous control β-actin expression. The normalized data of each gene were analyzed with 2^-ΔCt^ method. The experiments were done in triplicate for each sample. Sequences of the primers used for each gene are given in S1 Table.

### ROS measurement

ROS concentration in samples was measured using an OxiSelect Intracellular ROS Assay Kit (Cell Biolabs, San Diego, CA). The assay uses 2’,7’-dichlorodihydrofluorescin diacetate (DCFH-DA), which is deacetylated to non-fluorescent 2’,7’-dichlorodihydrofluorescin and then oxidized by ROS to highly fluorescent 2’,7’-dichlorofluorescin (DCF). Each of the samples were mixed with 1×DCFH-DA solution in a 96-well black plate and incubated at 37°C for 48 hrs. ROS concentration in the samples was measured by determining the fluorescence intensities of DCF at 480 nm using Spectra Max Gemini EM (Molecular devices, Sunnyvale, CA).

### Measurement of SOD activity

SOD activity in samples was determined using an OxiSelect Superoxide dismutase activity assay kit (Cell Biolabs). This assay uses a xanthine/XO system to produce superoxide anions, which reduce chromagen to produce a formazan dye, which is colorimetrically detectable at 490 nm. SOD activity in the samples was determined as the inhibition of formazan dye production. Each of the samples was mixed with 1× XO solution in a 96-well black plate and incubated at 37°C for 60 min and the formazan dye produced was colorimetrically detected at 490 nm using Spectra Max Plus (Molecular devices).

### PM preparation

After anesthesia with intramuscular injection of ketamine (Daiichi-Sankyo Propharma Co., LTD.), PM was obtained by inoculating 10 mL of cold PBS into the abdominal cavity by using 26-gauge needles attached to a 20-mL syringe. After a soft massage of the abdomen for 30 seconds, the peritoneal liquid was collected with 18-gauge needles attached to a 20-mL syringe. This procedure was repeated 2 times for each animal. Total peritoneal liquids were centrifuged at 300×g for 10 minutes to collect PM. The collected cells were adjusted to a concentration of 2.5×10^6^ cells/mL in Dullbecco’s modified Eagle’s medium (DMEM, Wako Pure Chemicals) supplemented with 10% FBS and cultured in a 96-well plate for 3 hrs. The cells were then cultured in DMEM medium containing 1mg/mL of control IgG, 38–2, 3S9 or 2H9 mAb together with DS or control DMSO for 3hrs before being subjected to Western blotting, ELISA, real-time RT-PCR, FACS, and immunoprecipitation followed by mass spectrometry.

### Alveolar macrophage preparation

Lungs were lavaged twice by injection with 2.0 ml of sterile saline using a syringe and the lavaged fluids were harvested by gentle aspiration. The fluids were then centrifuged at 300×g for 5 minutes to collect cells. The cells were adjusted to a concentration of 2.5×10^5^ cells/mL in DMEM supplemented with 10% FBS and cultured in a 96-well plate for 3hrs. The cells were then treated with 1mg/mL of control IgG or 38–2 mAb in DMEM supplemented with 10% FBS for 3hrs before being subjected to real-time RT-PCR.

### Construction of pIRES-hrGFP-MoPrP

The DNA fragment encoding full-length mouse PrP^C^ was amplified by PCR with a sense primer (5’-tcggatcccgtcatc**atg**gcgaac-3’; the underlined sequence, a *Bam*H I site; the bold sequence, a start codon) and an antisense primer (5’-ccctcgaggc**tca**tcccacgatcaggaag-3’; the underlined sequence, a *Xho* I site; the bold sequence, a stop codon) using pcDNA3.1/MoPrP plasmid encoding mouse PrP^C^ [44] as a template. After confirmation of the DNA sequences, each DNA fragment was digested by *BanH* I and *Xho* I and introduced into a pIRES-hrGFP-2a vector (Stratagene, La Jolla, CA).

### Flow cytometry

N2aΔPrP cells [26] were plated onto 6-well plates on the day before transfection. pIRES-hrGFP-MoPrP vector and control pIRES-hrGFP-2a vector (Stratagene) were transfected into N2aΔPrP cells using Lipofectamone 2000 (Invitrogen) according to the manufacturers’ instructions. The cells were harvested 72 hrs after transfection using PBS containing 20 mM EDTA and washed with FACS buffer (5% FBS in PBS). PM cells collected from the peritoneal cavity were washed with PBS, incubated with ACK lysing buffer (150 mM NH_4_Cl, 10 mM KHCO_3_, 0.1 mM EDTA) for 5 min to lyse red blood cells. The PM cells were then washed again with PBS and incubated with FACS buffer containing Fc-blocker (Biolegend, San Diego, CA; Cat. No. 101302) for 30 min on ice. Transfected N2aΔPrP and PM cells were incubated with 4 **μ**g/mL of control IgG (Sigma) or 38–2, 3S9, and 2H9 anti-PrP mAbs in the FACS buffer for 1 hr on ice. For PM cells, we also used PE anti-mouse/human CD11b antibody (Biolegend). The cells were washed with the FACS buffer 3 times to remove unbound antibodies, incubated with 1:800 diluted Alexa Fluor 647 anti-mouse antibody (Invitrogen), washed with PBS 3 times, suspended in PBS containing 10 mM EDTA and 0.2% bovine serum albumin, and analyzed using a CytoFLEX Flow Cytometer with CytExpert software, version 2.3 (Beckman Coulter, Inc., Brea, CA). Data were analyzed using FlowJo software, version 10.6.2 (Treestar Unc., Ashland, OR).

### Mass spectrometry analysis

PM were treated for 3 hrs in DMEM medium containing 1 mg/mL of control IgG or 38–2 mAb as described above, followed by two washes with ice-cold PBS. Cells were lysed in RIPA lysis buffer (1% NP-40, 0.25% Sodium deoxycholate, 0.05% SDS, 150 mM NaCl, 20 mM HEPES-NaOH, pH 7.5, 1 mM EGTA, and 1 mM MgCl_2_) containing Complete protease inhibitors (Merck Millipore, Darmstadt, Germany), PhosSTOP phosphatase inhibitors (Merck Millipore), and 50 units/ml of benzonase endonuclease　(Merck Millipore) on ice for 10 min. After centrifugation at 20,000×g for 15 min at 4°C, the supernatants were incubated with anti-phosphorylated SFK (Tyr416) antibody (Cell Signaling Technology) that was immobilized on SureBeads Protein G magnetic beads (Bio-Rad, Hercules, CA) for 3 hrs at 4°C with rotation. The beads were washed four times with RIPA buffer and then with 50 mM ammonium bicarbonate twice. Proteins on the beads were digested with 200 ng trypsin/Lys-C mix (Promega) for 16 hrs at 37°C. The digests were reduced, alkylated, acidified, and desalted using GL-Tip SDS (GL Sciences, Tokyo, Japan). The eluates were evaporated and dissolved in 3% acetonitrile (ACN) and 0.1% trifluoroacetic acid. LC-MS/MS analysis of the resultant peptides was performed on an EASY-nLC 1200 UHPLC (Thermo Fisher Scientific) connected to an Orbitrap Fusion mass spectrometer (Thermo Fisher Scientific) equipped with a nanoelectrospray ion source (Thermo Fisher Scientific). The peptides were separated on a 75 μm inner diameter ×150 mm C18 reversed-phase column (Nikkyo Technos, Tokyo, Japan) with a linear 4–32% ACN gradient for 0–100 min followed by an increase to 80% ACN for 10 min. The mass spectrometer was operated in a data-dependent acquisition mode with a maximum duty cycle of 3 sec. MS1 spectra were measured with a resolution of 120,000 and a mass range from 375 to 1,500 *m/z*. HCD MS/MS spectra were acquired in the linear ion trap with an isolation window of 1.6 *m/z* and a normalized collision energy of 30. Dynamic exclusion was set to 15 sec. Raw data were directly analyzed against the SwissProt database restricted to *M*. *musculus* using Proteome Discoverer version 2.4 (Thermo Fisher Scientific) with Mascot search engine version 2.5 (Matrix Science, London, UK). The search parameters were as follows: (i) trypsin as an enzyme with up to two missed cleavages; (ii) precursor mass tolerance of 10 ppm; (iii) fragment mass tolerance of 0.6 Da; (iv) carbamidomethylation of cysteine as a fixed modification; and (v) acetylation of the protein N-terminus and oxidation of methionine as variable modifications. Peptides and proteins were filtered at a false-discovery rate (FDR) of 1% using the percolator node and the protein FDR validator node, respectively. Label-free precursor ion quantification was performed using the precursor ions quantifier node, and normalization was performed such that the total sum of abundance values for each sample over all peptides was the same. The abundance for each protein was analyzed using Proteome Discoverer version 2.4 (Thermo Fisher Scientific).

### Statistical analysis

Survival rates were analyzed using the log-rank (Mantel-Cox) test (GraphPad Prism software package version 5.04). All other statistical analysis data were determined using a Student’s *t*-test (Microsoft EXCEL software, version 16.37).

## Supporting information

S1 Table List of primers used in RT-PCR with their DNA sequences and the number of PCR cycles. (DOCX)

S1 Fig 38–2 mAb protects against lethal infection with IAV/PR8 in mice. The survival rate (%, upper panel) and body weight loss (%, lower panel) of WT mice intraperitoneally administered with either the buffer alone (0 mg/mouse) and 0.2 mg/mouse and 0.5 mg/mouse of 38–2 mAb 1 day before intranasal infection with 200 IFU of IAV/PR8. Error bars, standard deviations (SD). *, p<0.05.
(TIF)

S2 Fig 38–2 mAb suppresses lung exudates in mice infected with IAV/PR8. Wet lung/ body weight (%) in mice treated control IgG- and 38–2 mAb at 0 (uninfected), 3, 5, and 8 dpi with 200 IFU of IAV/PR8. **, p<0.01.
(TIF)

S3 Fig 38–2 mAb suppresses lung epithelial cell apoptosis in mice infected with IAV/PR8. Western blotting for the AT1 cell marker podoplanin, the AT2 cell marker SP-C, the Clara cell marker CC10, pro-caspase 3, and the cleaved caspase 3 in lungs from control IgG- and 38–2 mAb-treated mice uninfected and at 3 and 5 dpi with 200 IFU of IAV/PR8. Signal densities of these molecules were combined with those in Fig 2D to statistically quantify the densities of each molecule. Actb, β-actin.
(TIF)

S4 Fig 38–2 mAb suppresses apoptosis in the lungs of mice infected with IAV/PR8. TUNEL staining of the lungs from control IgG- and 38–2 mAb-treated mice uninfected and at 3 and 5 dpi with 200 IFU of IAV/PR8. Bar, 0.5 mm.
(TIF)

S5 Fig DS abolishes the protective activity of 38–2 mAb in mice infected with IAV/PR8. The survival rate (%, upper panels) and body weight loss (%, lower panels) of WT mice intraperitoneally administrated with control IgG (left panels) and 38–2 mAb (right panel) together with 10 mg of DS 1 day before intranasal infection with 200 IFU of IAV/PR8. Error bars, SD. *, p<0.05.
(TIF)

S6 Fig PP2 but not imatinib abolishes the protective activity of 38–2 mAb in mice infected with IAV/PR8. The survival rate (%, upper panels) and body weight loss (%, lower panels) of WT mice intraperitoneally administrated with control IgG (left panels) and 38–2 mAb (right panel) together with 5 mg of PP2 **(A)** or 200 mg of imatinib **(B)** 1 day before intranasal infection with 200 IFU of IAV/PR8. Error bars, SD. *, p<0.05; **, p<0.01.
(TIF)

S7 Fig 38–2 mAb polarizes alveolar macrophages to a M2 phenoptype. Real-time PCR for M1-specific genes (TNF-α and INF-γ) and M2-specific genes (MGL1 and IL-10) in alveolar macrophages collected from the BALFs of WT mice 3 hrs after treatment with control IgG and 38–2 mAb (n = 3 in each group). **, p<0.01.
(TIF)

S8 Fig 38–2 mAb increased phosphorylated Lyn (Tyr416) and, to a lesser extent, phosphorylated Hck (Tyr416) in peritoneal macrophages. The abundance of each SFK in the immunoprecipitate with anti-phosphorylated SFK (Tyr416) Ab in peritoneal macrophages 3 hrs after treatment with control IgG and 38–2 mAb.
(TIF)

S9 Fig Therapeutic effects of 38–2 mAb against lethal infection with IAV/PR8. The survival rate (%, upper panel) and body weight loss (%, lower panel) of WT mice intraperitoneally administered with 38–2 mAb 5 days after intranasal infection with 200 IFU of IAV/PR8. Control IgG was similarly injected into WT mice 3 days after infection with 200 IFU of IAV/PR8. Error bars, SD.
(TIF)

S10 Fig 38–2, 3S9, 2H9 mAbs recognize PrP^C^ on Western blotting. Uncropped, full picture of Western blotting for PrP^C^ with 38–2, 3S9, 2H9 mAbs in the brains (Br) and lungs (Lg) from WT and *Prnp*^*0/0*^ mice in Fig 10A.
(TIF)
