# Supplementary material for: Prion protein signaling induces M2 macrophage polarization and protects from lethal influenza infection in mice
Source: PLoS Pathog. 2020 Aug 26;16(8):e1008823. doi: 10.1371/journal.ppat.1008823 (PMC7489546; doi:10.1371/journal.ppat.1008823)
Supplement: S1 Table — (DOCX) [file ppat.1008823.s001.docx]

**Supplementary Table 1. List of primers used in real-time PCR with their DNA sequences**

| **Gene** | **Primer** | **Sequence** |
| --- | --- | --- |
| IL6 | sense  antisense | 5’-CGG CCT TCC CTA CTT CAC AA-3’  5’-GGA TGG TCT TGG TCC TTA GC-3’ |
| TNF-α | sense  antisense | 5’-GCC TAT GTC TCA GCC TCT TC-3’  5’-GGA GGT TGA CTT TCT CCT GG-3’ |
| IFN-α | sense  antisense | 5’-TGG CTA GGC TCT GTG CTT TC-3’  5’-AGC TGC TGG TGG AGG TCA TT-3’ |
| IFN-γ | sense  antisense | 5’-CTT GGC TTT GCA GCT CTT CC-3’  5’-GCT CAT TGA ATG CTT GGC GC-3’ |
| MCP1 | sense  antisense | 5’-CCT GCT GTT CAC AGT TGC-3’  5’-GTC TGG ACC CAT TCC TTC-3’ |
| iNOS | sense  antisense | 5’-CCA AGC CCT CAC CTA CTT CC-3’  5’-CAC TTC GCA CAA AGC AGG GC-3’ |
| IFIT1  MxA  ARG1  MGL1  IL10  Actb | sense  antisense  sense  antisense  sense  antisense  sense  antisense  sense  antisense  sense  antisense | 5’-TTG ATC CAG AGC GAG CAG CT-3’  5’-AGG TAG ATC TGG GCT TCT GC-3’  5’-GGA AGA GCT CTG TGC TGG AA-3’  5’-CAA TTT CAG CAC CAG AGG GC-3’  5’-TGG CTT GCG AGA CGT AGA C-3’  5’-CTC CTC TGC TGT CTT CCC A-3’  5’-GCT TCG AAA AAG GGA TCA GTT CT-3’  5’-CCC AGT TCT TAA AGC CTT TCT CA-3’  5’-ATA CTG CTA ACC GAC TCC T-3’  5’-ATG GCC TTG TAG ACA CCT-3’  5’-GGA CTC CTA TGT GGG TGA CGA GG-3’  5’-GGG AGA GCA TAG CCC TCG TAG AT-3’ |
