# Supplementary figures and images for: Prion protein signaling induces M2 macrophage polarization and protects from lethal influenza infection in mice
Source: PLoS Pathog. 2020 Aug 26;16(8):e1008823. doi: 10.1371/journal.ppat.1008823 (PMC7489546; doi:10.1371/journal.ppat.1008823)

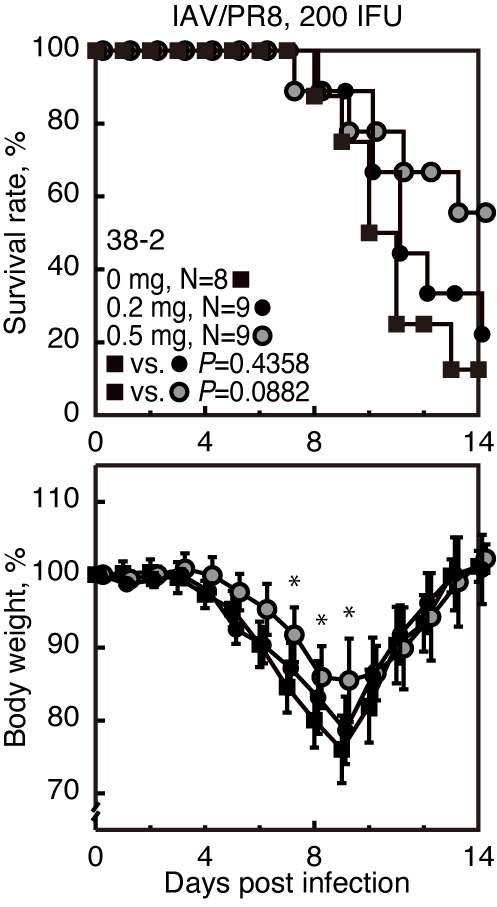

Supplement: S1 Fig — The survival rate (%, upper panel) and body weight loss (%, lower panel) of WT mice intraperitoneally administered with either the buffer alone (0 mg/mouse) and 0.2 mg/mouse and 0.5 mg/mouse of 38–2 mAb 1 day before intranasal infection with 200 IFU of IAV/PR8. Error bars, standard deviations (SD). *, p<0.05. (TIF) [file ppat.1008823.s002.tif]

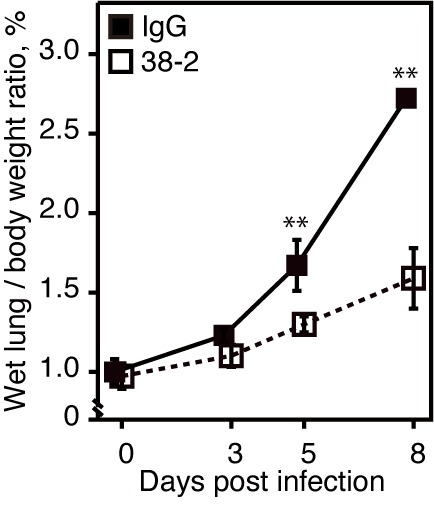

Supplement: S2 Fig — Wet lung/ body weight (%) in mice treated control IgG- and 38–2 mAb at 0 (uninfected), 3, 5, and 8 dpi with 200 IFU of IAV/PR8. **, p<0.01. (TIF) [file ppat.1008823.s003.tif]

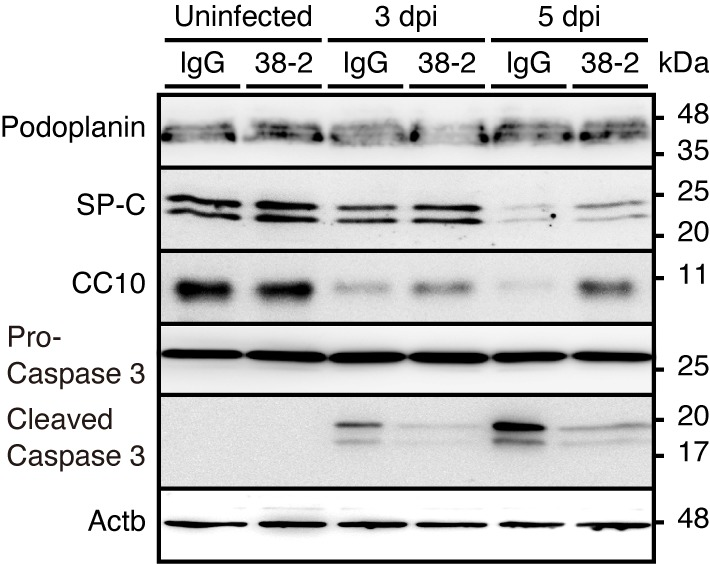

Supplement: S3 Fig — Western blotting for the AT1 cell marker podoplanin, the AT2 cell marker SP-C, the Clara cell marker CC10, pro-caspase 3, and the cleaved caspase 3 in lungs from control IgG- and 38–2 mAb-treated mice uninfected and at 3 and 5 dpi with 200 IFU of IAV/PR8. Signal densities of these molecules were combined with those in Fig 2D to statistically quantify the densities of each molecule. Actb, β-actin. (TIF) [file ppat.1008823.s004.tif]

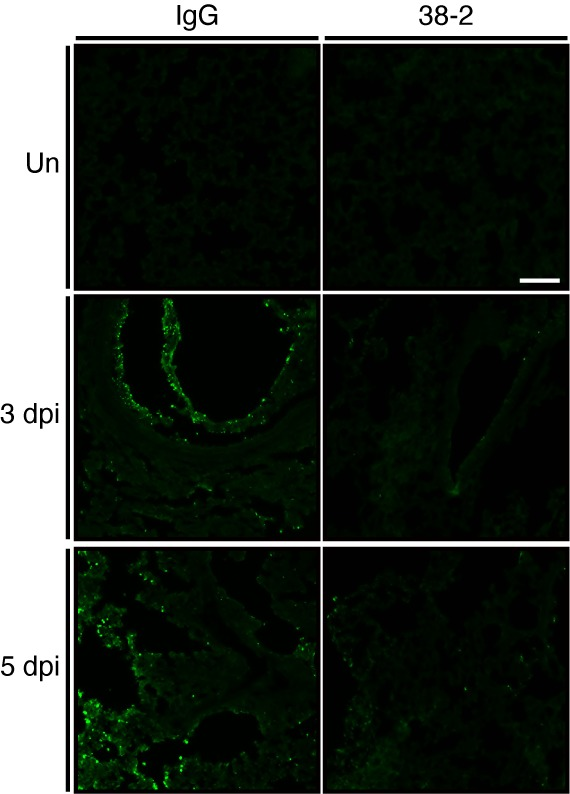

Supplement: S4 Fig — TUNEL staining of the lungs from control IgG- and 38–2 mAb-treated mice uninfected and at 3 and 5 dpi with 200 IFU of IAV/PR8. Bar, 0.5 mm. (TIF) [file ppat.1008823.s005.tif]

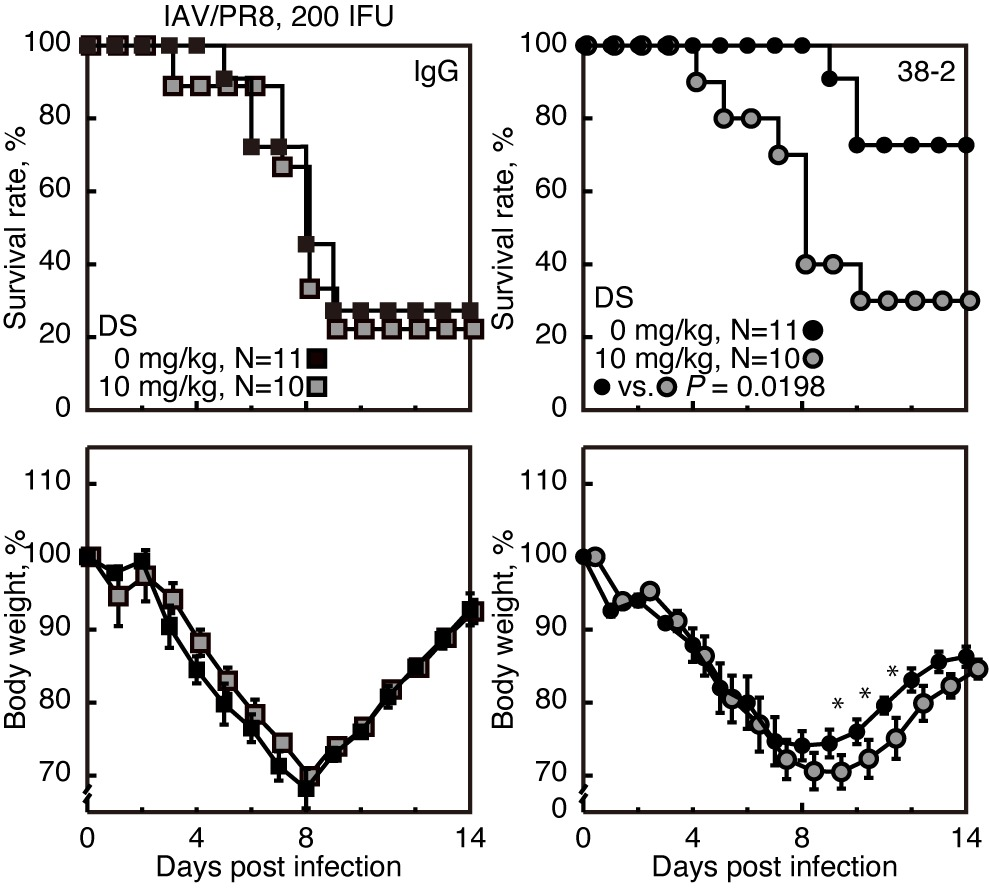

Supplement: S5 Fig — The survival rate (%, upper panels) and body weight loss (%, lower panels) of WT mice intraperitoneally administrated with control IgG (left panels) and 38–2 mAb (right panel) together with 10 mg of DS 1 day before intranasal infection with 200 IFU of IAV/PR8. Error bars, SD. *, p<0.05. (TIF) [file ppat.1008823.s006.tif]

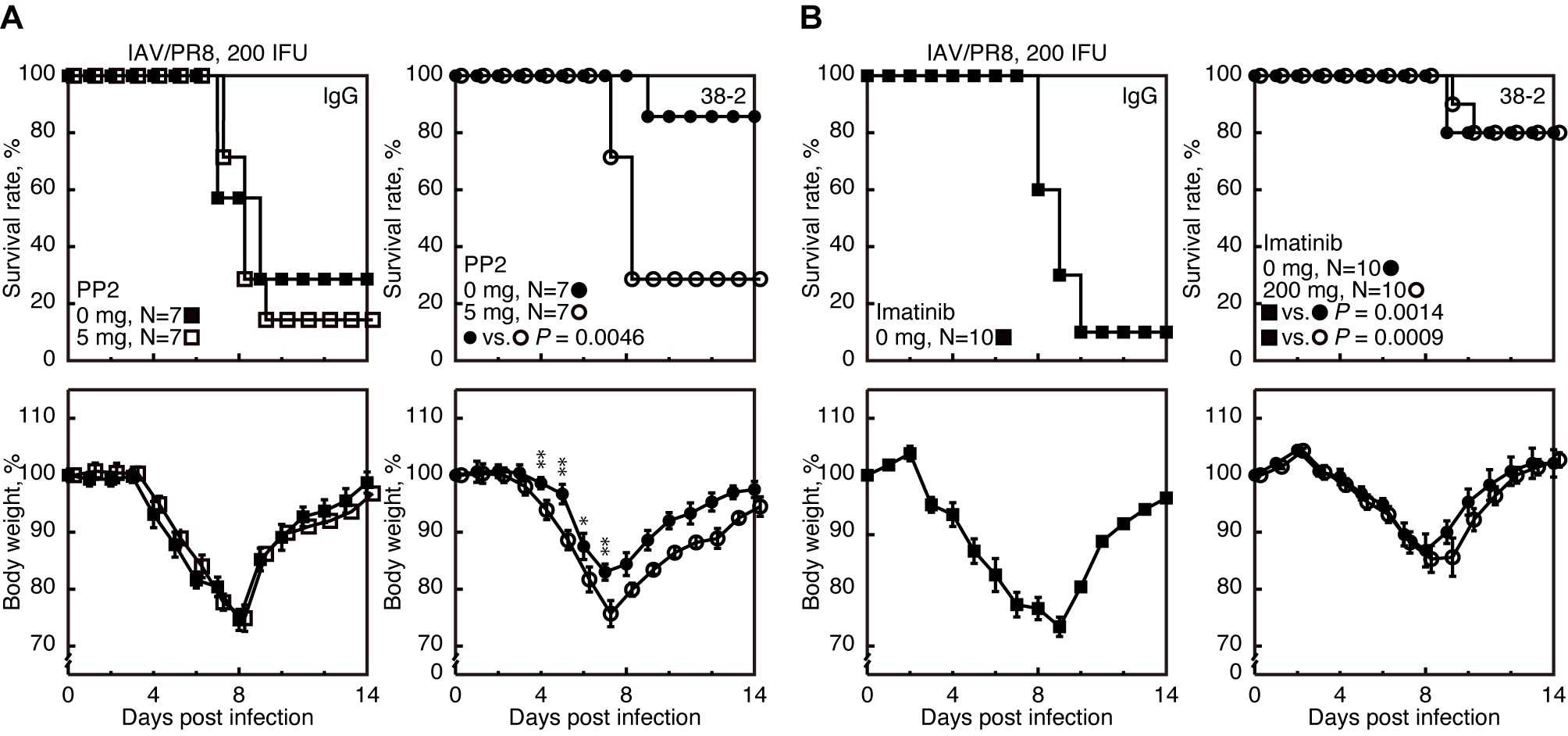

Supplement: S6 Fig — The survival rate (%, upper panels) and body weight loss (%, lower panels) of WT mice intraperitoneally administrated with control IgG (left panels) and 38–2 mAb (right panel) together with 5 mg of PP2 (A) or 200 mg of imatinib (B) 1 day before intranasal infection with 200 IFU of IAV/PR8. Error bars, SD. *, p<0.05; **, p<0.01. (TIF) [file ppat.1008823.s007.tif]

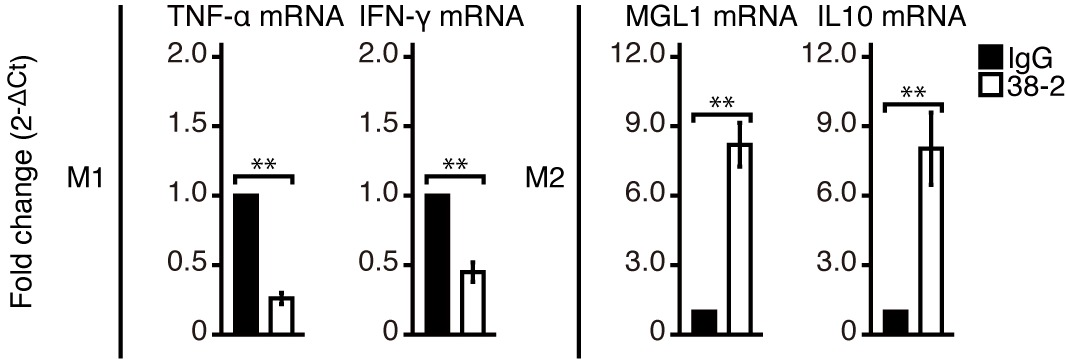

Supplement: S7 Fig — Real-time PCR for M1-specific genes (TNF-α and INF-γ) and M2-specific genes (MGL1 and IL-10) in alveolar macrophages collected from the BALFs of WT mice 3 hrs after treatment with control IgG and 38–2 mAb (n = 3 in each group). **, p<0.01. (TIF) [file ppat.1008823.s008.tif]

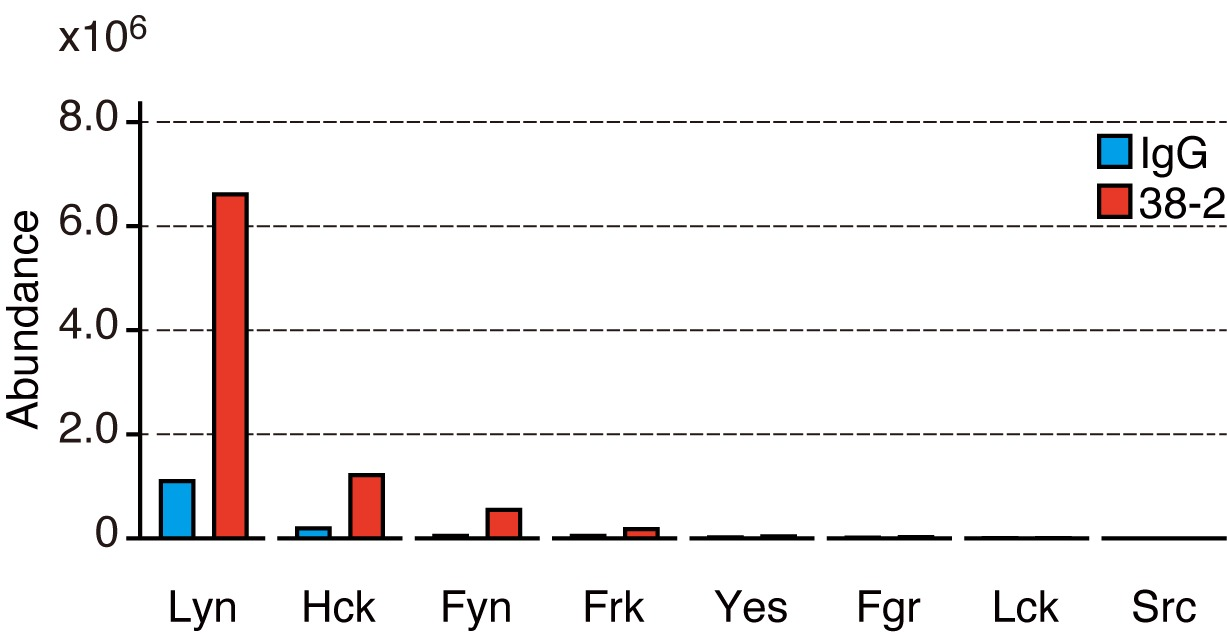

Supplement: S8 Fig — The abundance of each SFK in the immunoprecipitate with anti-phosphorylated SFK (Tyr416) Ab in peritoneal macrophages 3 hrs after treatment with control IgG and 38–2 mAb. (TIF) [file ppat.1008823.s009.tif]

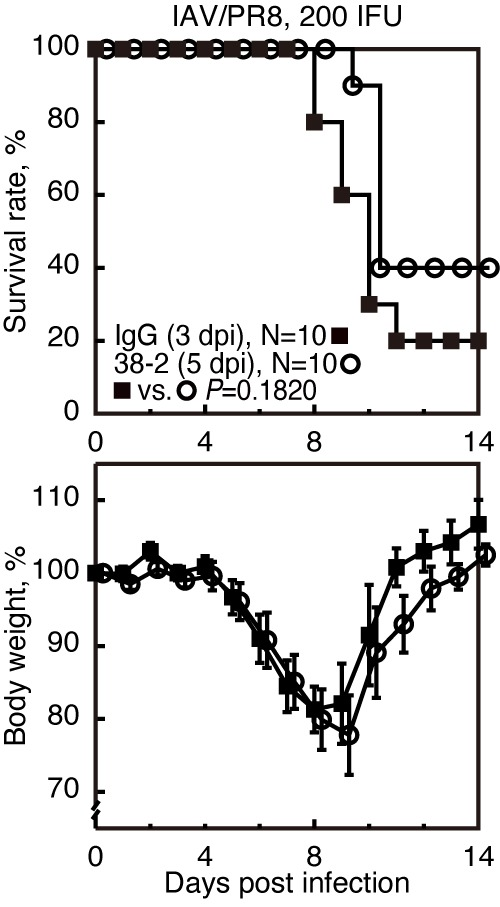

Supplement: S9 Fig — The survival rate (%, upper panel) and body weight loss (%, lower panel) of WT mice intraperitoneally administered with 38–2 mAb 5 days after intranasal infection with 200 IFU of IAV/PR8. Control IgG was similarly injected into WT mice 3 days after infection with 200 IFU of IAV/PR8. Error bars, SD. (TIF) [file ppat.1008823.s010.tif]

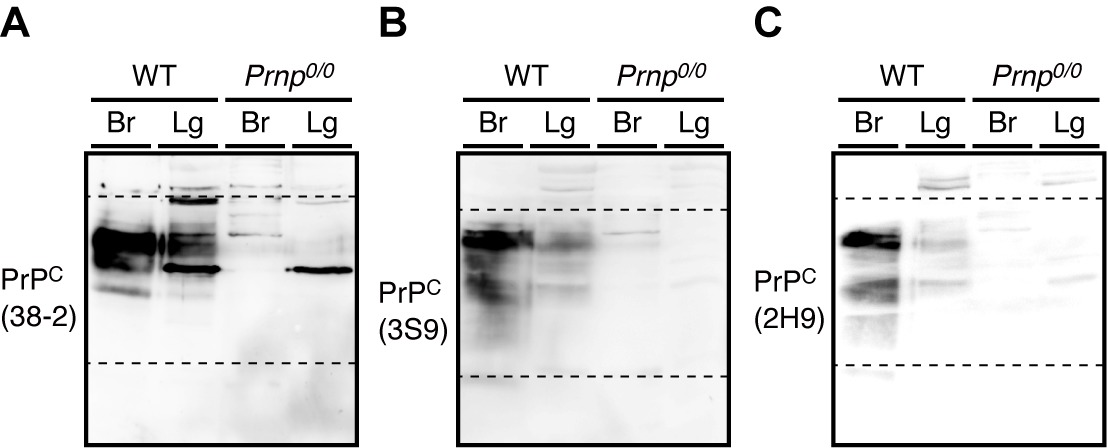

Supplement: S10 Fig — Uncropped, full picture of Western blotting for PrPC with 38–2, 3S9, 2H9 mAbs in the brains (Br) and lungs (Lg) from WT and Prnp0/0 mice in Fig 10A. (TIF) [file ppat.1008823.s011.tif]
